# Variation of N cycle guilds of the rye rhizosphere microbiome is driven by crop productivity along a tillage erosion catena

**DOI:** 10.1093/ismeco/ycaf020

**Published:** 2025-03-21

**Authors:** Simon Lewin, Marc Wehrhan, Sonja Wende, Michael Sommer, Steffen Kolb

**Affiliations:** Microbial Biogeochemistry, Research Area Landscape Functioning, Leibniz Centre for Agricultural Landscape Research e.V. (ZALF), 15374 Müncheberg, Germany; Julius Kühn Institute (JKI)—Federal Research Centre for Cultivated Plants, Institute for Epidemiology and Pathogen Diagnostics, Messeweg 11–12, 38104 Braunschweig, Germany; Landscape Pedology, Research Area Landscape Functioning, Leibniz Centre for Agricultural Landscape Research e.V. (ZALF), 15374 Müncheberg, Germany; Microbial Biogeochemistry, Research Area Landscape Functioning, Leibniz Centre for Agricultural Landscape Research e.V. (ZALF), 15374 Müncheberg, Germany; Landscape Pedology, Research Area Landscape Functioning, Leibniz Centre for Agricultural Landscape Research e.V. (ZALF), 15374 Müncheberg, Germany; Microbial Biogeochemistry, Research Area Landscape Functioning, Leibniz Centre for Agricultural Landscape Research e.V. (ZALF), 15374 Müncheberg, Germany; Thaer Institute, Faculty of Life Sciences, Humboldt University of Berlin, 10115 Berlin, Germany

**Keywords:** crop microbiome, remote sensing, denitrification, metagenomics, soil erosion, crop biomass patterns

## Abstract

Tillage erosion poses threats to crop yields. A transition towards more sustainable agricultural practices may be advanced by harnessing ecosystem services provided by plant microbiomes. However, targeting microbiomes at the agroecosystem scale necessitates bridging the gap to microscale structures of microbiomes. We hypothesized that differences of microbial nitrogen (N) cycle guilds in the rhizosphere of rye align with a soil catena that has been formed by tillage erosion. The rhizosphere was sampled at four sites, which captured a complete tillage erosion gradient from extremely eroded to depositional soils. The gene abundances characteristic of microbial N cycle guilds were assessed via metagenomics. The eroded sites showed the lowest plant productivity and soil mineral N availability, which was associated with an enrichment of *glnA* in the rhizosphere. Genes associated with dissimilatory nitrate-to-ammonium reducers and diazotrophy prevailed in the eroded soil profiles. The strongest correlations of the biomasses of rye plants along the catena with N cycle functions were observed for *norBC*. Thus, tillage erosion as a legacy of agricultural management aligns with substantial differences in rhizosphere microbiome functionality in N cycling. These microbiome differences were linked to plant shoot properties. Thus, the dynamics of the microbiome can be indirectly assessed by remote sensing.

## Introduction

Tillage erosion poses significant threats to crop yields [1, 2]. Balancing intensification and sustainability of agriculture involves the promotion and utilization of ecosystem functions provided by soil and plant microbiomes [3, 4]. However, a comprehensive understanding of microbiome-mediated ecosystem services and thus their spatial and temporal patterns at scales that are relevant to agroecosystems necessitates bridging the informational gap between microscale information of microbiomes at a spot and their variation at the field scale and beyond [5, 6]. Canonically, heterogeneous terrestrial ecosystems are often sampled along grids and transects, so that their environmental drivers and spatial heterogeneities can be captured systematically [7–10]. An alternative sampling approach would be to identify specific deterministic ecosystem elements that sufficiently capture variation of microbiome structure and functional traits. Hence, the dynamics and spatial patterns of soil microbiomes associated with aboveground crop productivity can be leveraged [11]. To reduce sampling efforts further, aboveground crop productivity can be easily assessed through remote sensing via the enhanced vegetation index (EVI) [12].

Tillage erosion represents a major mode of soil redistribution in arable fields, especially in hummocky ground moraine landscapes [13]. Soil redistribution by tillage erosion leads to a reduced top soil thickness at eroded terrain positions and a greater soil thickness at depositional positions. Thus, differences in root development, nutrient availability, and water storage occur, which ultimately leads to in-field crop productivity gradients along the erosion catena [12, 14]. Consequently, these changes affect the composition and distribution of soil microbiomes, inducing the selection of copiotrophs or oligotrophs [9, 15, 16].

Rather than taxonomic microbial groups, microbial guilds are likely more similar across space and time [17, 18]. Thus, we expected that along an erosion catena the distribution of microbial guilds—i.e. microorganisms utilizing and producing the same resource—will be even more stratified than the taxonomic microbiota composition [1]. Specifically, the microbiota involved in N cycling depend on plant productivity since both plants and microbial N cycle guilds rely on N compounds [19]. An investment of the plant into their associated shorter-lived rhizosphere microbiome ultimately increases N availability to the plant [20]. The spatial organization of microbial N cycle guilds has been examined in grassland soils and arable fields [5, 8, 10]. Microbial N cycle guilds are defined as microorganisms involved in the same N transformations and are thus subdivided into denitrifiers, dissimilatory nitrate-to-ammonium reducers (DNRAs), nitrifiers, and diazotrophs as well as those that degrade and assimilate organic N compounds. The association of microbial N cycle guilds with in-field plant productivity is exceptionally important since these guilds release and assimilate N. Estimates of crop N demand rely on shoot total N content (STN) [21, 22].

We aimed to perform a comprehensive assessment of in-field plant growth variation and its correlation with the distribution patterns of dissimilatory and assimilatory microbial N cycling guilds. We hypothesized that differences in soils that led to varying shoot biomass would also result in distinct rhizosphere microbiome functions in winter rye plants. We thus expected that microbial N cycle guilds associated with denitrification and organic N compound degradation would be more abundant at non-eroded slope and colluvic soils than at eroded sites. Conversely, we hypothesized that microbial N cycle guilds involved in N assimilation and the provision of inorganic N, such as DNRAs and diazotrophs, would be more abundant in the rhizosphere of soils with poor plant growth, which is characteristic of strongly eroded soils.

We followed characteristic differences in soils along a tillage erosion catena to capture substantial changes in microbiomes. The rationale for doing so was rooted in the assumption that the functional microbial variation at the field scale would be driven by changes in crop productivity along the erosion catena. Thus, no sampling along a linear transect was needed. We specifically targeted the microbial N cycle guilds based on metagenomic reads, which were annotated to genes encoding enzymes involved in the transformation of N compounds.

## Materials and methods

### Sampling along an erosion catena

**Figure 1:**
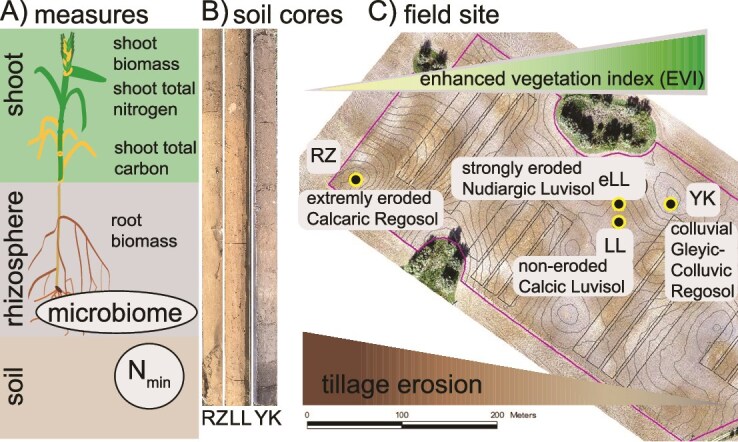
Survey of microbial N cycle guild distribution along soils affected by tillage erosion: the rhizosphere of rye plants was sampled along a catena typically exhibiting characteristic differences of shoot biomass. (A) Overview of the experimental measures. (B) Soil cores of soils sampled (3/4). (C) Four soils subjected to soil redistribution by tillage erosion were sampled: extremely eroded hilltop (RZ) > strongly eroded slope (eLL) > non-eroded slope (LL) > depositional sink (YK). Plant biomass decreases typically from YK to RZ characterized by a decreasing enhanced vegetation index.

For our study, we selected a representative field (11 ha) grown with winter rye (2021) at the landscape laboratory ‘AgroScapeLab Quillow’ of Leibniz Centre for Agricultural Landscape Research (ZALF) (NE Brandenburg, Germany). The Quillow catchment is characterized by a hilly topography with short summit–foot–slope distances [12]. This topographic pattern is depictive of ground moraine landscapes, which developed during the retreat of the Weichselian glaciers (ca. 15 ka BP) [23]. The region’s climate is classified as subcontinental and summarized by a mean temperature and precipitation of 9.1°C and 505 mm, respectively (yearly average 1992–2022, Deutscher Wetterdienst [DWD] meteorological station, Grünow), and an average precipitation during the winter wheat and rye growing season of 306 mm (monthly average 1992–2022, DWD meteorological station, Grünow). We investigated four soils representing a full gradient along a tillage erosion catena [24] and captured local variation at each site by sampling four positions in a 4 × 4-m^2^ square (Fig. 1). The soils were classified as extremely eroded Calcaric Regosol (RZ), strongly eroded Nudiargic Luvisol (eLL), non-eroded Calcic Luvisol (LL), and colluvial Gleyic-Colluvic Regosols (YK) on the basis of 1-m soil cores drilled in March 2021 (Additional Fig. S1). At RZ, the glacial till (C horizon) starts right below the plough layer at 0.3 m (Ap). At eLL, the C horizon starts at 0.6 m (Bt still present), whereas LL comprises a full horizon sequence for Luvisols (Ap-E-Bt-C) with C depths >1 m. The YK is characterized by an accumulation of (former) topsoil from the catchment down to 1 m. In the lower part, groundwater influence can be recognized by redoximorphic features. Accordingly, soil organic carbon (SOC), TN, TC, and phosphorus (P) showed a characteristic pattern (Additional Fig. S1). All four properties are lower in the topsoil of RZ than in that of LL and are highest for YK. The subsoil of YK shows highest SOC, TN, TC, and P. The soil pH ranges from 6.5 to 7.9 with the highest values at RZ due to carbonates.

To relate the in-field pattern of the aboveground crop biomass of rye to the EVI, two unmanned air vehicle (UAV) flights were conducted pre-flowering (BBCH 52–55; 17 May 2021) and post-flowering (BBCH 75–75; 16 June 2021). Ground sampling campaigns (plant biomass, topsoil) were performed at four 0.25-m^2^ patches within each 4 × 4-m plot. The aggregated bulk soil was removed from the root system by shaking. Aboveground and belowground plant biomass were separated and root systems with adhering rhizosphere were transferred to the laboratory while cooling on dry ice. The root systems were washed with phosphate buffered saline (PBS) buffer (pH 7.4) and shaken on a rotary shaker (125 rpm, 5 min) to separate the rhizosphere from roots. The detached soil suspension was then centrifuged at 4500×*g* for 10 min to collect the rhizosphere in pellets. All samples were stored at −80°C until further processing.

### Soil and plant chemical parameters

All analyses were conducted by ZALF’s central laboratory facility in cooperation with sample preparation by ZALF’s field station. Soil moisture (SM) and soil nitrate (soil NO_3_^−^) and ammonium (soil NH_4_^+^) were measured from bulk soil samples extracted with KCl using a photometric flow through analyser (CFA-SCAN; Skalaranalytic GmbH, DIN ISO 14256). Aboveground plant biomass was weighed and oven dried to analyse shoot fresh weight (SFW) and shoot dry weight (SDW). A subsample was ground to analyse shoot total nitrogen (STN) and shoot total carbon (STC) by combustion (CNS928-MLC; Leco Instruments GmbH) according to DIN ISO 13878 and 10 694, respectively.

### Remote sensing

Multispectral imagery were captured by a Micasense RedEdge-MX sensor, mounted on a fixed-wing UAV (Trinity F90+; Quantum Systems GmbH, Gilching, Germany). The sensor covers the spectral range from visible (VIS) to near infrared (NIR) wavelength in five discrete wavebands (blue: 475 nm, green: 560 nm, red: 668 nm, red edge: 717 nm, near infrared: 840 nm). Flight missions were carried out on 17 May and 14 June 2021 under clear sky conditions. The flight altitude of 100 m above ground resulted in a spatial resolution of 0.07 m. Images were pre- and post-processed according to a previously published procedure using Agisoft Metashape Professional software [25]. A widely used method in remote sensing is the calculation of vegetation indices from VIS and NIR reflectance as proxies for biophysical vegetation quantities. In this study, we used the EVI [26] (Equation 1) motivated by studies reporting a trend to more linear relationships with aboveground biomass of crops and a wider range of values at the same time than other commonly used indices such as the normalized difference vegetation index [27, 28].

**Equation 1**: G, gain factor; C1, C2, coefficients of the aerosol resistance term; L, soil background reflectance adjustment (G = 2.5, C1 = 6, C2 = 7.5, and L = 1); Near infrared, Red, and Blue, calibrated reflectance values of the respective wavebands.


\begin{align*} EVI=G\times \frac{Near\ infrared- Red\ }{Near\ infrared+C1\times Red-C2\times Blue}+L \end{align*}

### Deoxyribonucleic acid extraction

Genomic DNA was obtained from 0.4 g of the rhizosphere pellet using the DNeasy PowerLyzer PowerSoil Kit (QIAGEN GmbH, Hilden, Germany) according to the manufacturer’s instructions. The lysis step was performed with a FastPrep-24 bead beater at a speed of 3400 rpm (MP Biomedicals, LLC, Goddard, Irvine, CA, USA). A Qubit assay (Invitrogen, Waltham, MA, USA) was used to measure the initial DNA concentration. The DNA extracts were diluted to uniform DNA concentrations and stored at −80°C until further analyses.

### Metagenomic shotgun sequencing and bioinformatics

Metagenomic sequencing of total extracted DNA from 31 rhizosphere samples was performed by CeGaT GmbH (Tübingen, Germany) on the Illumina NovaSeq 6000 platform in paired-end mode with a read length of 150 bp. Samples were sequenced to an average amount of 79G per sample (= 262.9 million reads pairs per sample). Trimmomatic v.0.39 [29] was used for adapter removal and read quality trimming. Reads were clipped if mean quality score dropped <20 in a sliding window of four. Only reads with lengths >75 bases were retained. Samples were filtered for host DNA by mapping reads to two rye reference genomes (GCA_016097815.1 and GCA_902687465.1) using the bowtie2 module in kneaddata v0.7.2. The concatenated forward and reverse reads were aligned to NCycDB [30] using diamond blastx (v2.0.15) [31] with settings -max-target-seqs 1 and -evalue 1e-4. On average, 3.12 million reads per sample were aligned to NCycDB target sequences. To provide a taxonomic profile of N cycling genes, all aligned reads were classified using kraken2 [32] with a microbial reference database.

### Statistics

All statistical analyses were conducted using R 4.2.1 [33]. Visualizations were produced by ggplot2 [34]. Sequencing read counts of metagenomic feature abundances were normalized by total sum scaling. Transformation-based redundancy analyses were performed using the vegan package [35] to explore multivariate effects while applying a log transformation on metagenomics feature abundances. The associations of both categorical predictors and continuous predictors on metagenomics feature abundances were assessed using the workflow of microbiome multivariable associations with linear models (MaAslin) [36]. The models that still conflict with the parametric model after data transformation were excluded from the analyses. Differences amongst sites and correlations of environmental parameters were assessed using linear mixed models from lme4 [37] with growth stage as a random factor. Conflicts with linear model and parametric test assumptions were verified by statistical tests and visual inspection implemented in the performance package [38] (outlier check, linear relationship of predictors and the response, normality of the residuals and random effects, heteroscedasticity). *Post hoc* pairwise comparisons, coefficients, and effect sizes were based on marginal means and calculated using the emmeans package [39] and fundamental data records (FDR) corrections for multiple testing were applied. Random forest analyses were performed with the mlr3 package and the random forest learner implemented in the ranger package [40]. The model was evaluated using leave-one-out cross-validation. Variable selection was based on permutation importance. The differential abundance of taxonomic abundances derived from the metagenomic reads assigned to N cycling genes was performed using linear discriminant analyses effect size (LEfSe) [41]. This tool delineates biomarker taxa, which are characteristic of predefined statistical classes, here the soils along the catena.

## Results

### Correlation of the enhanced vegetation index with crop productivity measures

The EVI was recorded during two UAV missions conducted on 17 May and 16 June. These ground measures were calibrated to the EVI using linear mixed model regression (Additional Fig. S2). The strongest correlations were obtained for STN followed by plant STC and SFW. Only a moderate correlation occurred for SDW.

### Effects of soil horizon profiles on plants and edaphic parameters

Four terrain positions along a tillage erosion catena were surveyed. The hilltop (RZ), two slopes (LL and eLL), and a depression (YK) were selected because of their characteristic differences in crop aboveground biomass. In accordance with this *a priori* selection, the SFW increased significantly along the catena at the pre-flowering stage from the lowest value at the hilltop to maximum value at the depression (Table 1, Additional table S1), whereas at post-flowering stage LL and YK were not discernible. STN was significantly higher at both slopes and at the depression compared to the hilltop. This difference was even most evident at post-flowering. Thus, the characteristic crop productivity gradient from RZ to YK along the erosion catena based on measurements of STN, SFW, and SDW was confirmed, so that the *a priori* selection was justified. However, the eroded slope had the highest SDW at post-flowering.

**Table 1:** Effect of soils (ELL, LL, RZ, YK) on rye shoot and soil parameters. Matrix of effects (lower triangular) and *P* values (upper triangular, bold significant <.05) of differences^a^ between hilltop (RZ), eroded slope (eLL), slope (LL), and depression (YK) for shoot fresh weight (SFW), shoot dry weight (SDW), shoot total carbon (STC), shoot total nitrogen (STN), rhizosphere soil mineral ammonium (soil NH_4_^+^), rhizosphere soil mineral nitrate (soil NO_3_^−^), and rhizosphere soil moisture.

	**eLL**	**LL**	**RZ**	**YK**	**eLL**	**LL**	**RZ**	**YK**
**RZ**	SFW	.89	**.02**	.82	SFW	.16	**.00**	.89
**eLL**	116.00	SFW	**.02**	.69	619.00	SFW	.06	.20
**LL**	2162.00	2047.00	SFW	**.01**	1493.00	874.00	SFW	**.00**
**YK**	−183.00	−298.00	−2345.00	SFW	59.00	−560.00	−1434.00	SFW
**RZ**	SDW	.28	.12	.22	SDW	**.03**	**.01**	**.04**
**eLL**	−119.70	SDW	**.01**	.86	357.30	SDW	.47	.91
**LL**	178.40	298.10	SDW	**.01**	465.90	108.60	SDW	.41
**YK**	−138.10	−18.40	−316.50	SDW	340.20	−17.10	−125.70	SDW
**RZ**	STC	.63	.69	SDW	STC	.9265	.2509	**.0234**
**eLL**	−.10	STC	.93	.07	.0200	STC	.2178	**.0279**
**LL**	−.08	.02	STC	.08	−.2560	−.2760	STC	**.0025**
**YK**	.28	.38	.36	STC	.5510	.5310	.8070	STC
**RZ**	STN	.8302	**.0408**	.5682	STN	.50120	**.02300**	**.00260**
**eLL**	−.0307	STN	**.0192**	.6980	−.05250	STN	**.00640**	**.00940**
**LL**	.3236	.3542	STN	.0094	.19730	.24980	STN	<.0001
**YK**	−.0822	−.0515	−.4057	STN	−.28630	−.23380	−.48350	STN
**RZ**	Soil NH_4_^+^	.71720	.05450	.82880	Soil NH_4_^+^	.07	.28	**<.0001**
**eLL**	−.00094	Soil NH_4_^+^	**.01980**	.53490	−.0054	Soil NH_4_^+^	**.0089**	**<.0001**
**LL**	.00544	.00638	Soil NH_4_^+^	.06120	.0030	.0084	Soil NH_4_^+^	**<.0001**
**YK**	.00056	.00150	−.00488	Soil NH_4_^+^	−.0346	−2.93E-02	−3.76E-02	Soil NH_4_^+^
**RZ**	Soil NO_3_^−^	.83	.38	.88	Soil NO_3_^−^	.16	**.00**	.88
**eLL**	−.01	Soil NO_3_^−^	.25	.95	.22	Soil NO_3_^−^	**.01**	.13
**LL**	.04	.05	Soil NO_3_^−^	.27	.67	.45	Soil NO_3_^−^	**.00**
**YK**	−.01	.00	−.05	Soil NO_3_^−^	−.02	−.25	−.69	Soil NO_3_^−^
**RZ**	Soil.moist.	.0648	.1720	.5643	Soil.moist.	**.0460**	**<.0001**	**<.0001**
**eLL**	.8860	Soil.moist.	.5366	**.0156**	−.4810	Soil.moist.	<.0001	.7280
**LL**	.6310	−.2550	Soil.moist.	**.0484**	1.9230	2.4040	Soil.moist.	<.0001
**YK**	−.2570	−1.1430	Soil.moist.	Soil.moist.	−.4040	.0770	−2.3270	Soil.moist.

a Contrasts: RZ-eLL, RZ-LL, RZ-YK, eLL-LL, eLL-YK, LL-YK

The soil ammonium content was significantly greater at eLL than at the hilltop. The soil NH_4_^+^ content was also significantly higher at the depression than in all other soils at post-flowering growth stage. The soil moisture was significantly greater at the depression than in the hilltop and the non-eroded slope at the pre-flowering stage. However, the soil moisture was lowest at the hilltop at post-flowering. Hence, such transient edaphic parameters differed along the catena.

### Abundances of microbial N cycle guilds

The relative abundance of genes involved in microbial N cycling were obtained by metagenomics (Additional Fig. S3). These genes were categorized as follows: energy metabolism, organic N compound synthesis, and degradation pathways of organic N compounds (Additional Table S2). The majority of operons (average 53.7%) were associated with organic N compound synthesis, followed by N assimilation (average 14.2%) and organic N compound degradation (average 14.9%) (Additional Fig. S3). Further genes of diazotrophs accounted for 0.03% on average.

The most abundant energy metabolism operons belonged to denitrification (average 10.6%) (Additional Fig. S3). These genes were twice as abundant as genes indicating DNRA (average 5.1%) and 70-fold enriched compared to those of nitrification (average 0.15%). The denitrifiers were dominated by the *nirK* type, which was 4-fold more abundant than *nirS* type and was characterized by an overall *nir* to *nosZ* ratio of 1.53 (Additional Fig. S4). The DNRA guild contained 16-fold more fermentative (*nirBD*) than respiratory (*nrf*) DNRA genes. Approximately 10 times more archaeal (AOA) than bacterial (AOB) ammonia oxidizers constituted the nitrification guild, while AOA outnumbered nitrite oxidizers by a factor of 1.5 (Additional Fig. S4). The AOA:AOB ratio changed significantly along the erosion catena, with AOA being between 15 and 20 times more abundant than AOB at eroded soil. In turn, AOA and AOB were almost equally abundant at YK.

### Soil erosion states and abundances of microbial N cycle guilds

The differential abundance of N cycling genes averaged at the operon level (Additional Table S2) was examined to identify significantly enriched microbial N cycle guilds along the erosion catena (Supplementary text 1, Additional Table S3, Additional Fig. S3). Differences between both growth stages were marginal. Thus, further analyses were performed independently of the growth stage.

We aimed to identify operons, which substantially contribute to distinguishing rhizosphere microbiomes of the four soils. Two random forest models were fitted to classify the operon abundances into the four soils. The first model relied on the operons, which belong to dissimilatory microbial N cycling guilds and the model had an aggregated prediction accuracy 0.90. The second model had an aggregated prediction accuracy 0.87 and used the operons, which belong to microbial N cycling guilds in charge of N assimilation and organic N compound turnover as input features. Thus, the models were trained and evaluated to learn patterns in microbial N cycling guilds abundances, which were most characteristic to the four soils.

**Figure 2:**
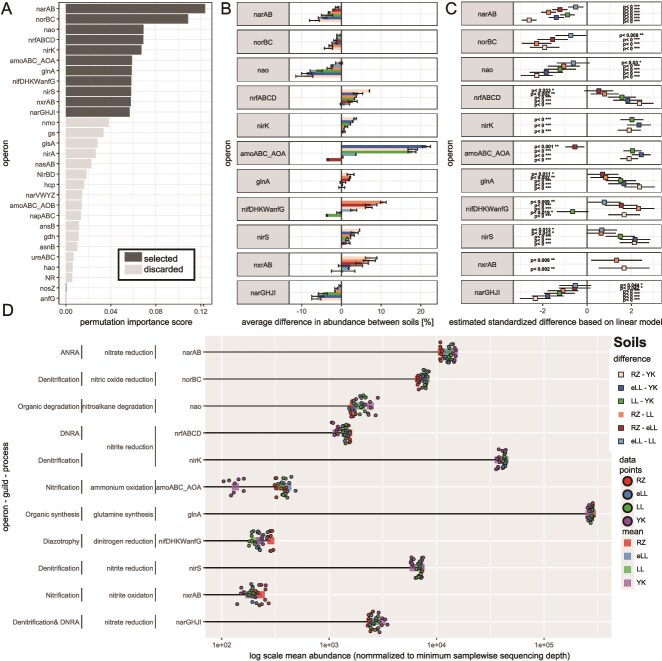
Characteristic difference in abundances of microbial N cycling guilds of soils along the erosion catena. (A) Permutation importance of random forest classifications of the four soils.^1^ (B) Average relative difference in abundances of operons selected by random forest between soils.^2^ (C) Standardized estimated differences in abundances and confidence intervals of operons selected by random forest between soils based on analysis of variance (ANOVA) of linear models and *post hoc* Tukey tests corrected for multiple comparisons. (D) Lollipop chart^1^ of individual abundances (*n* = 8) and mean abundances per soils. ^1^Segment drawn to maximum value. ^2^Top 11 operons considered for further analyses based on two models using either operons, which belong to dissimilatory process or assimilation and synthesis, or degradation of organic N as input features. ^3^Normalized to total counts per operon.

*NarAB* and *nif* (assimilatory), *glnA* (organic N synthesis), *nao* (organic N degradation), and seven dissimilatory operons contributed most to the model performance as evident from a shift in permutation feature importance (Fig. 2A). Thus, these 11 operons discriminated the rhizosphere microbiomes along the erosion catena. The differences in abundances of a single operon were typically most pronounced between eroded (RZ, eLL) compared to the depression (YK) and non-eroded soil (LL). However, *nif* and *amoABC* (AOA) did not follow this pattern, although they showed the greatest relative differences across all the operons selected by the random forest model.

*NorB* and *narAB* had the greatest importance in the random forest model performance, but showed comparably small relative differences between soils*.* Only *narGHJI* and *narAB* differed significantly in abundance between all four soils, whereas *nxrAB* was significantly different only between RZ compared to LL and YK. The enrichment showed large relative differences between soils (Fig. 2B, C), and their contribution to the random forest performance was moderate. Compared with RZ, *amoABC* (AOA) was enriched at eLL. *nif* was more abundant at LL than at YK. These enrichment patterns do not follow the direction of the catena development in contrast to the other operons identified by the random forest models.

### Soil- and plant-associated environmental drivers

Overall, the microbial N cycle guild composition exhibited a strong correlation with soil- and plant-associated environmental drivers as indicated by the redundancy analysis (RDA) models (Fig. 4). Accordingly, sites were always clustered by terrain positions, with the most pronounced clustering observed for assimilation guilds at the pre-flowering and degradation guilds at post-flowering stage. Notably, the eroded and non-eroded slope soils (eLL and LL) were not well distinguished.

**Figure 3:**
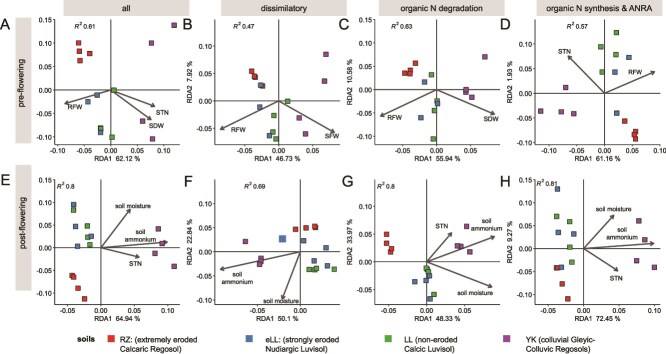
RDA analyses of N cycling gene compositions. Significant RDA models were optimized by forward and backward optimization.

At the pre-flowering stage (Fig. 3A–D), shoot and root biomass as well as STN determined the composition of all three categories of microbial N cycle guilds, whereas post-flowering STN, soil ammonium content, and soil moisture were identified as the most influential factors. In particular, the depositional site (YK) was separated from all others (Fig. 3E–H).

As a result, STN, which was correlated with the EVI and two edaphic properties altered by tillage erosion, namely, soil ammonium content and soil moisture, predicted a substantial proportion of variation in microbial N cycle guild composition. Notably, microbial functional guilds involved in organic N compound transformations had stronger effect sizes than dissimilatory guilds. The latter are likely strongly affected by site-specific boundary conditions that also determine plant biomass patterns.

**Figure 4:**
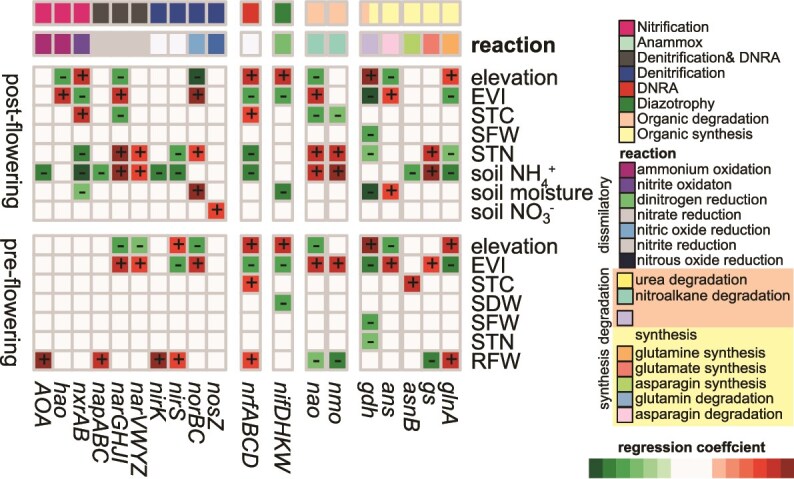
Response of the abundances of microbial N cycle guilds to plant and soil parameters. Relative gene abundances were summarized at the operon level. The colour scale only highlights the coefficient of models with *R^2^* >0.5. Model linear mixed model formula: gene abundance ~ environmental driver with plant growth stage as random factor.

The corresponding gene abundances were averaged at the operon level (Additional Table S5) and filtered to regression models with an *R*^2^ value >0.5. Regardless of the plant growth stage, elevation and EVI were the most prevalent predictors (Fig. 4). Therefore, these correlations support the hypothesis that the *a priori* selected soils cover a substantial amount of variation in the abundances of microbial N cycle guilds. Strong correlations were found between EVI (pre-*gdh R*^2^ 0.87; post-*narAB R*^2^ 0.90) and elevation (pre-*narAB R*^2^ 0.84; post-*gdh*, *narAB R*^2^ 0.88) with the relative abundances of certain genes. Soil ammonium content (*gs R*^2^ 0.90; *nxrAB R*^2^ 0.86) and soil moisture (gdh *R*^2^ 0.87) after flowering also showed to be strongly correlated with specific gene abundances.

Most regressions of environmental variables with unknown spatial patterns, unlike EVI and elevation, were identified at the post-flowering stage. Soil NH_4_^+^ content predicted the abundances of almost all functional genes included in the analyses (Fig. 4). STN was also a predictor for the relative abundance of multiple genes of different N cycle guilds. These genes were involved in the nitrite oxidation, the dissimilatory nitrite reduction, and organic N compound degradation. STC was exclusively affiliated with genes of assimilatory or degradation pathways. RFW occurred as predictor only at pre-flowering (Fig. 4). Our analyses indicated covariance of soil ammonium content with distinct correlations of DNRA and denitrifiers to STN. RFW predicted the relative abundances of genes participating in glutamine and glutamate assimilation and nitroalkane degradation. Moreover, RFW formed only negative correlations with genes of dissimilatory metabolisms. Amongst these were only *nirK*, but not *nirS*, nitrate reduction via *nap* genes, and archaeal ammonia oxidizers.

In summary, the EVI and elevation largely predicted individual abundances of microbial N cycling metabolic pathways of the rhizosphere microbiome of rye. Both factors covaried with the effect of the soils. Edaphic and shoot parameters were scarcely correlated at the growth stage pre-flowering, while most statistical associations were identified at post-flowering.

### Biomarker taxa analyses

Metagenomic reads successfully mapped to NCycDB were also classified taxonomically separately for each microbial N cycle guild. Subsequently, a differential abundance analysis using LEfSe was conducted to support the functional analyse of nitrification, diazotrophy, and organic N degradation guilds (Supplementary text 2, Supplementary Figs. S3–S5).

## Discussion

A comprehensive assessment of the functional dynamics of crop microbiomes in the rhizosphere is crucial for their integration into agricultural management. We focussed here on the various microbial N cycle guilds, which are crucial to the N availability of the crop plant [5]. Our study demonstrated that the functions of rhizosphere microbiomes in organic N degradation, uptake, and catabolic conversion aligns with an in-field gradient of cereal crop productivity and soil types along a tillage erosion catena. Notably, such identification of variations in the functions of rhizosphere microbiomes is not limited to tillage erosion. Furthermore, modulating factors, such as short- and long-term changes in fertilization, SOC content, mineralogy, and topography [1, 6, 42, 43], as well as vegetation properties directly affected by soil microbiomes, may also be relevant for the distribution of microbiome functionality [44]. Therefore, understanding the systematic dependence of crop productivity on soil development allows to grasp the functions of crop microbiomes at a scale that is relevant to agroecosystem functioning [8]. The latter is of utmost importance for leveraging ecosystem services realized by the crop microbiome such as promoting plant-beneficial effects, controlling diseases, and reducing greenhouse gas emissions [45, 46]. We speculate that field-scale tracking of crop microbiomes by observing systematic variation in aboveground plant productivity, as demonstrated here for a tillage erosion catena, can support farming solutions that aim to maintain the multitude of ecosystem services provided by soil microbiomes.

The EVI serves as a proxy for overall plant productivity to predict STN and STN [11, 12]. Therefore, predicting belowground root and rhizosphere microbiome traits and functions via proxies would allow for mapping the distribution of microbiome functions, [47] and would help to understand the impact of soil microbiomes on aboveground ecosystem functions [48].

The influence of plant traits and growth differences has been examined in diverse grassland plant communities [49]. Differences in aboveground plant biomass and STN at the regional to landscape scale are the result of distinct plant growth strategies or traits. Thus, these methods are suitable for predicting microbial biogeography in grasslands [49]. We aimed to establish a similar relationship for rye grown as a monoculture and found that the variation in mineral N content in the rhizosphere was tied to the variation in STN, STC, and EVI through nitrate-utilizing and nitrate-producing microbial N cycle guilds. The variation in plant shoot properties is usually considered to reflect their organic N nutrition state [48]. Multiple microbial metabolisms, including the degradation of organic N compounds and nitrate reduction (encoded by *narGHJI* or *narAB*), exhibited strong interrelationships with aboveground plant productivity and soil ammonium content. Thus, the differences in shoot properties of rye observed along the soil erosion catena in our study likely reflected the distinct microbial N cycle guild abundances.

Plant nitrate uptake and the subsequent release of carbonate can directly influence microbial N cycle guilds involved in the utilization and production of nitrate [50]. The transient dynamics of the rhizosphere microbiome of annual crops observed here may contribute to long-term changes in soil pH. Soil pH is well known to determine microbial biogeography at regional, landscape, and continental scales [49, 51, 52]. How and to what degree changes in the rhizosphere microbiome translate into altered bulk soil pH and other soil properties such as the SOC requires further investigations.

Rolling landscapes exhibit higher levels of denitrification activity and an increased presence of putative denitrifiers at foot slopes [1, 45, 53]. *nirK* and *nirS* were crucial indicators for classifying the rhizosphere microbiome into the four soils examined, likely reflecting ecological niche separation between *nirK*- and *nirS*-type denitrifiers. Increased intermicrobial competition at the SOC-rich sites LL and YK likely favours *nirK*-type denitrifiers [8, 54]. Additionally, tillage practices directly affect the niche partitioning of *nirK* and *nirS*. Consistent with previous findings [11], we concluded that *nirK*-type denitrifiers were enriched in the rhizosphere of highly productive rye plants. On the other hand, *nirS*-type denitrifiers prevailed in rye plants with low productivity, as indicated by the negative correlations observed in this study between *nirS* abundances and EVI and STN. While the distribution of *nirS*-type denitrifiers is known to be driven by differences in soil pH and nitrate content, *nirK*-type denitrifiers are affected by complex factors such as tillage and soil structure [8] and are relatively enriched in rhizosphere soil [11].

*NorBC* abundance was strongly correlated to soil moisture and *NorBC* had high utility in discriminating the four soils. Indeed, ambiguous abundances of N cycling genes along a soil catena depend on soil drainage, and associated biomass patterns rely on seasonal changes in precipitation [7, 12]. Soil water availability strongly affect plant and microbiome activity and functions. Drought and intense precipitation will increase in frequency due to climate change and will affect the abundances of plant-beneficial microbiota in crop production systems [55]. The *norBC* operon is involved in the regulation of auxin biosynthesis carried out by plant growth–promoting bacteria and participates in canonical denitrification and nitrifier denitrification [56, 57]. Thus, *norBC* abundance likely responded to a multitude of potential plant–microbiome–soil interactions before and after flowering. We conclude that future research should address changes in *norBC* abundance in interaction with water stresses to better understand the impact of drought and flooding on crop microbiomes.

The genes associated with either DNRA or denitrification exhibited opposing distribution patterns along the soil erosion catena. These findings contrasted with previously reported higher abundances of DNRA under no tillage [58]. *nrfABCD* was correlated with plant productivity (EVI, STN) and soil ammonium content. Unlike denitrification genes, the abundance of *nrfABCD* was correlated with STC. This correlation implied a stronger dependence of DNRA on the carbon allocation of plants than on that of denitrifiers, which is corroborated by the fact that DNRA predominates over denitrifiers at high C:N ratios [59, 60]. DNRA is regulated by both nitrite and nitrate concentrations and may also depend on nitrous oxide, which underpins the relevance of *norBC* [59, 61].

The nitrite derived from *napAB* is subsequently utilized by the nitrite reduction to ammonium through an oxidoreductase that is encoded by *nrfABCD*. Moreover, *napAB* activity is thought to be modulated by the plant nitrate transporter NRT1.1b, which is sensitive to nitrate in the rhizosphere [62]. This finding is consistent with previous findings that reported the predominance of DNRA bacteria in the rhizosphere and detritusphere [63, 64]. Thus, the aboveground to belowground associations of the *nap* and *nrf* pathway may indicate that the rye plant actively recruited DNRA-competent microbiota to the rhizosphere. Remarkably, DNRA bacteria predominated only at the nutrient-poor eroded sites. We speculate that plant species–specific mechanisms to recruit ammonium-preserving microorganisms might have been involved since plants benefit in the long term from competition for N with their microbiome [20].

Our analyses revealed covariance of the soil ammonium content with distinct correlations of DNRA and denitrifiers with STN. N uptake and allocation of N in cereals alternate after flowering, which is reflected by STN [65]. Whether plant nitrate transporters drive the competition and niche occupation between DNRA and specific denitrifiers (*NirS* or *NirK*) in the rhizosphere requires further validation. The relationship between plant N uptake and recruitment of DNRA or denitrification to the rhizosphere can be assessed by transcriptomic analyses of microbial guilds in combination with root windows, which allows the observation of rhizodeposition in the field [66].

The high abundance of nitrite oxidizers at the eroded site (RZ) was reflected by both the soil (soil moisture, ammonium content) and the aboveground plant biomass parameters (EVI, STN, STC) as well as showed high predictive power in the random forest model. Nitrite oxidizers (NOB) showed a similar distribution pattern as AOA. This suggests co-occurrence of AOA and NOB [5]. However, based on our metagenome-based taxonomic classification, typical taxa known as NOB were identified as a LEfse biomarker of the eroded site (RZ). Siderophore production by *Nitrospira* species that may oxidize nitrite or are confounded by comammox species can be beneficial for both plant and ammonium oxidizer growth [66]. Urea-hydrolysing NOB or AOA can promote plant growth by mineralizing soil organic matter (SOM) in nutrient-poor eroded soils [67, 68]. This explanation is also applicable to our findings, as we observed an abundant increase of *ureABC* at the eroded site (RZ).

Diazotrophs can provide additional N to plants, also as free-living types [69, 70]. *nif* abundance was amongst the highly predictive features in the random forest classification. Free-living diazotrophs, such as *Geobacter*, were LEfSe biomarkers of that site in our study. *Geobacter* diazotrophs can be associated with switchgrass and are correlated with crop productivity [71]. Moreover, diazotrophs can be enriched in the rhizosphere of rye at sites with low N availability and being strongly eroded [6, 16]. The abundance of *nif* genes was correlated with EVI pre-flowering and SDW post-flowering. Thus, we were able to confirm the dependence of the *nif* gene abundance on plant productivity as previously reported [6, 71]. Therefore, our findings provide further evidence for the hypothesis that cereal crops acquire diazotrophs as an adaptation strategy under N limitation [6, 72, 73]. However, other drivers of *nif* abundance appeared to exist, which distinguished non-eroded and colluvic soils.

Two indicator genes of nitroalkane (*nmo*) and nitronate degradation (*nao*) were most abundant at depositional site. Both operons were positively correlated with the EVI, STN, and soil ammonium content and negatively correlated with the STC and RFW. Fungi synthesize 3-nitropropionate, and both bacteria and fungi use *nmo* genes during the detoxification in accordance with the LEfSe biomarkers of the depositional site (YK) [74]. This selection might eventually affect soil mineral N levels and plant productivity. These cascades are essential for the assembly of the entire rhizosphere microbiome [73].

In contrast, microbial N cycle guilds in charge of ammonium assimilation into amino acids (*glnA*, *gdh*) were enriched at the eroded site (RZ). The *glnA:gdh* ratio can be considered as a proxy for delineating N availability in an environment, with *glnA* being favoured during N limitation [64]. Therefore, the observed enrichment of *glnA* at the eroded site (RZ) and its utility in the random forest classification of the four soils corroborate our conclusion that the rhizosphere microbiome at the eroded site (RZ) experienced N limitation, which might have favoured the acquisition of DNRA bacteria and diazotrophs. Both *napAB* and *glnA* abundances were correlated with RFW at pre-flowering. The soil ammonium content and STN were also correlated via *gdh* and *glnA* abundances. Therefore, we speculated that changes in N assimilation and N conversion induced by DNRA and diazotrophs resulted from plant belowground sensing of nitrate and subsequently altered rhizodeposition [63].

Our study revealed that the N cycle–associated functions of rhizosphere microbiomes change along a tillage erosion catena characterized by an in-field gradient of aboveground plant productivity. In our study, the abundances of the denitrification, DNRA, diazotroph, and ammonium assimilation microbial guilds were strongly associated with aboveground plant productivity parameters and the EVI. The use of stable associations between plant productivity and local soil types is thus a promising approach for mapping rhizosphere microbiome functions at agroecosystem scales.

## Supplementary Material

Supplementary_Material_Additional_Text_Tables_and_Figures_ycaf020

## Data Availability

The sequence datasets generated and analysed during the current study are available in the NCBI Sequence Read Archive repository as project PRJNA1125628. All other data generated or analysed during this study are included in this published article and its supplementary information files.
