# Supplementary material for: Variation of N cycle guilds of the rye rhizosphere microbiome is driven by crop productivity along a tillage erosion catena
Source: ISME Commun. 2025 Mar 21;5(1):ycaf020. doi: 10.1093/ismeco/ycaf020 (PMC11931286; doi:10.1093/ismeco/ycaf020)
Supplement: Supplementary_Material_Additional_Text_Tables_and_Figures_ycaf020 [file supplementary_material_additional_text_tables_and_figures_ycaf020.docx]

Supplementary material

Variation of N cycle guilds of the rye rhizosphere microbiome is driven by crop productivity along a tillage erosion catena

**Simon Lewin^1,3,4^, Marc Wehrhan^2^, Sonja Wende^1^, Michael Sommer^2^, Steffen Kolb^1,3*^**

^1^Microbial Biogeochemistry, Research Area Landscape Functioning, Leibniz Centre for Agricultural Landscape Research e.V. (ZALF), Müncheberg, Germany

^2^Landscape Pedology, Research Area Landscape Functioning, Leibniz Centre for Agricultural Landscape Research e.V. (ZALF), Müncheberg, Germany

^3^Thaer Institute, Faculty of Life Sciences, Humboldt University of Berlin, Berlin, Germany

^4^ Current address: Julius Kühn Institute (JKI)-Federal Research Centre for Cultivated Plants, Institute for Epidemiology and Pathogen Diagnostics, Messeweg 11–12, 38104 Braunschweig, Germany

[*kolb@zalf.de](mailto:*kolb@zalf.de)

## Supplementary text 1 - results

Differential abundance of N cycling genes - averaged at the operon level (**Additional table 2**) - was examined to identify significantly enriched microbial N cycle guilds along the erosion catena (**Additional table 3**). AOB were significantly enriched at YK compared to all other sites with the strongest difference persisting between YK and LL. Contrary, AOA were least abundant at YK. Nitrite reducer were more abundant at RZ compared to LL and YK with the effect size increasing in that order. The diazotrophs were least abundant at the slope positions and significantly enriched at RZ, which was most pronounced post-flowering.

Both nitrite reducer groups (*nirS*, *nirK*) were significantly decreased at the depositional site (YK). The *nirS:nirK* ratio (**Additional figure 4**) was significantly increased at YK and to a lesser extent at LL compared to RZ. Nitric oxide reducers were most abundant at the slopes and significantly enriched compared to RZ. *nosZ* was significantly more abundant at the slopes compared to both YK and RZ (**Additional table 3**).

The abundances of *nrf* and *nap*, representing the respiratory DNRA, were least abundant at YK and most abundant at the slopes. In contrast to respiratory DNRA, *nirBD* associated with assimilative and dissimilatory nitrite reduction was most abundant at LL. and significantly contrasted from RK and YK post-flowering. However, *nirBD* does not follow the same trends as *narGHJI* which is involved in nitrate reduction to nitrite. Instead, *narGHJI* abundance increased gradually along the catena, which was most evident at pre-flowering.

Nitroalkane degradation (genes of operons *nmo* and *nao*) was overall significantly enriched at YK. The slopes were significantly enriched in glutamine and asparagine degradation. Glutamate degradation or assimilation by bidirectional *gdh* and assimilation by *glnA* was most strongly associated with RZ. *glnA* and *asn* abundance were significantly decreased at YK compared to the LL, eLL and RZ, whereby the highest abundances persisted at eLL and RZ. In turn, glutamate synthesis (*gs*) was was highest at YK compared to all other soils (**Additional table 3**). Both, *glnA* and *gdh* were most abundant at RZ. The *gdh:glnA*-ratio (**Additional Figure 4**) indicated that the N assimilation to glutamine and glutamate at YK was realized by *gdh*, while *glnA* predominated at YK at pre-flowering.

## Supplementary text 2 - results

**Biomarker taxa analyses**

Metagenomic reads successfully mapped to NCycDB were also classified taxonomically separately for each microbial N cycle guild. Subsequently, a differential abundance analyses using LEfSe was conducted. Regarding nitrification, the class *Nitrospira* was a biomarker of RZ based on *nxrAB,* while the class Betaproteobacteria was a biomarker of eLL based on *amoABC* (Additional figure 3). Prominent biomarker of diazotrophy were *Geobacter* at RZ post flowering and multiple Betaproteobacteria at LL (pre-flowering) and YK (post-flowering) (**Additional figure 4**).

Due to the cosmopolitan role of organic N degradation, a vast amount of taxa was differentially abundant between the soils along the erosion catena. We focus here on *nao* and *nmo*, since their functional abundances were also discriminated between sites and were correlated with environmental variables. Most remarkably, *Streptomyces*, *Pseudomonas* and *Burkholderia* were enriched at YK (**Additional figure 5**). Moreover, Ascomycota were exclusively found as a biomarker taxon based on *nmo* reads among all N degradation pathways.

## Supplementary tables

**Additional table 1**: ANOVA effect of soils on shoot and soil properties.

|  | **Df** | **Sum.Sq** | **Mean.Sq** | **F.value** | **p.value** | **response** | **growth.stage** |
| --- | --- | --- | --- | --- | --- | --- | --- |
| soil.type | 3 | 887876 | 295959 | 4.366 | **0.0296** | SFW | pre-flowering |
| Residuals | 11 | 745615 | 67783 |  |  | SFW | pre-flowering |
| soil.type | 3 | 261218 | 87073 | 1.403 | 0.2899 | SFW | post-flowering |
| Residuals | 12 | 744957 | 62080 |  |  | SFW | post-flowering |
| soil.type | 3 | 15886 | 5295 | 4.379 | **0.0293** | SDW | pre-flowering |
| Residuals | 11 | 13302 | 1209 |  |  | SDW | pre-flowering |
| soil.type | 3 | 34667 | 11556 | 4.383 | **0.0266** | SDW | post-flowering |
| Residuals | 12 | 31639 | 2637 |  |  | SDW | post-flowering |
| soil.type | 3 | 0.00004 | 0.00001 | 1.772 | 0.2105 | STC | pre-flowering |
| Residuals | 11 | 0.00008 | 0.00001 |  |  | STC | pre-flowering |
| soil.type | 3 | 0.45511 | 0.15170 | 1.000 | 0.4263 | STC | post-flowering |
| Residuals | 12 | 1.82092 | 0.15174 |  |  | STC | post-flowering |
| soil.type | 3 | 0.01714 | 0.00571 | 4.422 | **0.0285** | STN | pre-flowering |
| Residuals | 11 | 0.01421 | 0.00129 |  |  | STN | pre-flowering |
| soil.type | 3 | 1.21273 | 0.40424 | 1.078 | 0.3953 | STN | post-flowering |
| Residuals | 12 | 4.50043 | 0.37504 |  |  | STN | post-flowering |
| soil.type | 3 | 0.00001 | 0.00000 | 2.914 | 0.0821 | soil.ammonium | pre-flowering |
| Residuals | 11 | 0.00001 | 0.00000 |  |  | soil.ammonium | pre-flowering |
| soil.type | 3 | 0.00022 | 0.00007 | 82.889 | **0.0000** | soil.ammonium | post-flowering |
| Residuals | 12 | 0.00001 | 0.00000 |  |  | soil.ammonium | post-flowering |
| soil.type | 3 | 0.00692 | 0.00231 | 0.647 | 0.6009 | soil.nitrate | pre-flowering |
| Residuals | 11 | 0.03924 | 0.00357 |  |  | soil.nitrate | pre-flowering |
| soil.type | 3 | 2.85931 | 0.95310 | 1.325 | 0.3120 | soil.nitrate | post-flowering |
| Residuals | 12 | 8.63156 | 0.71930 |  |  | soil.nitrate | post-flowering |
| soil.type | 3 | 3.30068 | 1.10023 | 3.442 | 0.0555 | soil.moisture | pre-flowering |
| Residuals | 11 | 3.51642 | 0.31967 |  |  | soil.moisture | pre-flowering |
| soil.type | 3 | 15.29206 | 5.09735 | 54.484 | **0.0000** | soil.moisture | post-flowering |
| Residuals | 12 | 1.12268 | 0.09356 |  |  | soil.moisture | post-flowering |
| ^1^ shoot fresh weight (SFW), shoot dry weight (SDW), shoot total carbon (STC), shoot total nitrogen (STN), rhizosphere soil mineral ammonium (soil NH4+), rhizosphere soil mineral nitrate (soil NO3 ­-), and rhizosphere soil moisture (SM). | | | | | | | |

**Additional table 2**: N cycle gene annotations used to aggregate counts of sequences as curated in NCycDB.

| **Process** | **Pathway** | **Gene** | **Enzyme** | **Operon** |
| --- | --- | --- | --- | --- |
| Nitrification | ammonia oxidation | *amoA*_A | Ammonia monooxygenase subunit A (archaea) | *amoABC_Archaea* |
| Nitrification | ammonia oxidation | *amoB*_A | Ammonia monooxygenase subunit B (archaea) | *amoABC_Archaea* |
| Nitrification | ammonia oxidation | *amoC*_A | Ammonia monooxygenase subunit C (archaea) | *amoABC_Archaea* |
| Nitrification | ammonia oxidation | *amoA*_B | Ammonia monooxygenase subunit A (bacteria) | *amoABC_Bacteria* |
| Nitrification | ammonia oxidation | *amoB*_B | Ammonia monooxygenase subunit B (bacteria) | *amoABC_Bacteria* |
| Nitrification | ammonia oxidation | *amoC*_B | Ammonia monooxygenase subunit C (bacteria) | *amoABC_Bacteria* |
| Nitrification | ammonia oxidation | *hao* | Hydroxylamine dehydrogenase | *hao* |
| Nitrification | nitrite oxidation | *nxrA* | Nitrite oxidoreductase, alpha subunit | *nxrAB* |
| Nitrification | nitrite oxidation | *nxrB* | Nitrite oxidoreductase, beta subunit | *nxrAB* |
| Denitrification | nitrate reduction | *napA* | Periplasmic nitrate reductase NapA | *napABC* |
| Denitrification | nitrate reduction | *napB* | Cytochrome c-type protein NapB | *napABC* |
| Denitrification | nitrate reduction | *napC* | Cytochrome c-type protein NapC | *napABC* |
| Denitrification | nitrate reduction | *narG* | Nitrate reductase | *narGHIJ* |
| Denitrification | nitrate reduction | *narH* | Nitrate reductase | *narGHIJ* |
| Denitrification | nitrate reduction | *narJ* | Nitrate reductase molybdenum cofactor assembly chaperone | *narGHIJ* |
| Denitrification | nitrate reduction | *narI* | Nitrate reductase gamma subunit | *narGHIJ* |
| Denitrification | nitrite reduction | *nirK* | Nitrite reductase (NO-forming) | *nirK* |
| Denitrification | nitrite reduction | *nirS* | Nitrite reductase (NO-forming) | *nirS* |
| Denitrification | nitric oxid reduction | *norB* | Nitric oxide reductase subunit B | *norBC* |
| Denitrification | nitric oxid reduction | *norC* | Nitric oxide reductase subunit C | *norBC* |
| Denitrification | Nitrous-oxide reduction | *nosZ* | Nitrous-oxide reductase | *nosZ* |
| Denitrification | nitrate reduction | *narZ* | Nitrate reductase 2, alpha subunit | *narVWYZ* |
| Denitrification | nitrate reduction | *narY* | Nitrate reductase 2, beta subunit | *narVWYZ* |
| Denitrification | nitrate reduction | *narV* | Nitrate reductase 2, gamma subunit | *narVWYZ* |
| Denitrification | nitrate reduction | *narW* | Nitrate reductase 2, delta subunit | *narVWYZ* |
| Assimilatory nitrate reduction | nitrate reduction | *nasA* | Assimilatory nitrate reductase catalytic subunit | *nasAB* |
| Assimilatory nitrate reduction | nitrate reduction | *nasB* | Assimilatory nitrate reductase electron transfer subunit | *nasAB* |
| Assimilatory nitrate reduction | nitrite reduction | *nirA* | Ferredoxin-nitrite reductase | *nirA* |
| Assimilatory nitrate reduction | nitrate reduction | *NR* | Nitrate reductase (NAD(P)H) | *NR* |
| Assimilatory nitrate reduction | nitrate reduction | *narB* | Assimilatory nitrate reductase | *narBC* |
| Assimilatory nitrate reduction | nitrate reduction | *narC* | Cytochrome b-561 | *narBC* |
| Dissimilatory nitrate reduction | nitrate reduction | *napA* | Periplasmic nitrate reductase NapA | *napABC* |
| Dissimilatory nitrate reduction | nitrate reduction | *napB* | Cytochrome c-type protein NapB | *napABC* |
| Dissimilatory nitrate reduction | nitrate reduction | *napC* | Cytochrome c-type protein NapC | *napABC* |
| Dissimilatory nitrate reduction (fer) | nitrate reduction | *narG* | Nitrate reductase | *narGHIJVWYZ* |
| Dissimilatory nitrate reduction | nitrate reduction | *narH* | Nitrate reductase | *narGHIJVWYZ* |
| Dissimilatory nitrate reduction | nitrate reduction | *narJ* | Nitrate reductase molybdenum cofactor assembly chaperone | *narGHIJVWYZ* |
| Dissimilatory nitrate reduction | nitrate reduction | *narI* | Nitrate reductase gamma subunit | *narGHIJVWYZ* |
| Dissimilatory nitrate reduction | nitrate reduction | *narZ* | Nitrate reductase 2, alpha subunit | *narGHIJVWYZ* |
| Dissimilatory nitrate reduction | nitrate reduction | *narY* | Nitrate reductase 2, beta subunit | *narGHIJVWYZ* |
| Dissimilatory nitrate reduction | nitrate reduction | *narV* | Nitrate reductase 2, gamma subunit | *narGHIJVWYZ* |
| Dissimilatory nitrate reduction | nitrate reduction | *narW* | Nitrate reductase 2, delta subunit | *narGHIJVWYZ* |
| Dissimilatory nitrate reduction | nitrite reduction | *nirB* | Nitrite reductase (NADH) large subunit | *nirBD* |
| Dissimilatory nitrate reduction | nitrite reduction | *nirD* | Nitrite reductase (NADH) small subunit | *nirBD* |
| Dissimilatory nitrate reduction | nitrite reduction | *nrfA* | Nitrite reductase (cytochrome c-552) | *nrfABCD* |
| Dissimilatory nitrate reduction | nitrite reduction | *nrfB* | Cytochrome c-type protein NrfB | *nrfABCD* |
| Dissimilatory nitrate reduction | nitrite reduction | *nrfC* | Protein NrfC | *nrfABCD* |
| Dissimilatory nitrate reduction | nitrite reduction | *nrfD* | Protein NrfD | *nrfABCD* |

**Additional table 3:** Differential abundance of N cycle gene abundance summarized at the operon level based on linear models and estimate marginal means between soils along the erosion catena *(.xlsx)*

| **Differential abundance of N cycle gene abundances summarized at the operon level based on linear models and estimate marginal means** | | | | | | | | | |
| --- | --- | --- | --- | --- | --- | --- | --- | --- | --- |
| **contrast** | **estimate** | **SE** | **df** | **lower.CL** | **upper.CL** | **t.ratio** | **p.value** | **operon** | **growth stage** |
| RZ - eLL | -4.1E-01 | 1.7E-01 | 23 | -9.1E-01 | 8.4E-02 | -2.4E+00 | 3.0E-02 | amoABC_AOA | pre |
| RZ - YK | 2.1E+00 | 1.6E-01 | 23 | 1.6E+00 | 2.5E+00 | 1.3E+01 | 1.4E-11 | amoABC_AOA | pre |
| eLL - LL | 6.7E-01 | 1.7E-01 | 23 | 1.8E-01 | 1.2E+00 | 3.9E+00 | 1.0E-03 | amoABC_AOA | pre |
| eLL - YK | 2.5E+00 | 1.7E-01 | 23 | 2.0E+00 | 3.0E+00 | 1.4E+01 | 3.3E-12 | amoABC_AOA | pre |
| LL - YK | 1.8E+00 | 1.6E-01 | 23 | 1.3E+00 | 2.3E+00 | 1.1E+01 | 1.5E-10 | amoABC_AOA | pre |
| RZ - YK | -1.8E+00 | 4.8E-01 | 23 | -3.2E+00 | -4.0E-01 | -3.7E+00 | 3.5E-03 | amoABC_AOB | pre |
| eLL - YK | -2.0E+00 | 5.2E-01 | 23 | -3.5E+00 | -5.2E-01 | -3.9E+00 | 3.5E-03 | amoABC_AOB | pre |
| LL - YK | -1.7E+00 | 4.8E-01 | 23 | -3.1E+00 | -2.8E-01 | -3.5E+00 | 4.2E-03 | amoABC_AOB | pre |
| RZ - eLL | 1.4E+00 | 4.7E-01 | 23 | 2.5E-02 | 2.7E+00 | 2.9E+00 | 1.5E-02 | nxrAB | pre |
| RZ - LL | 1.9E+00 | 4.4E-01 | 23 | 6.9E-01 | 3.2E+00 | 4.5E+00 | 1.1E-03 | nxrAB | pre |
| RZ - YK | 1.5E+00 | 4.4E-01 | 23 | 1.9E-01 | 2.7E+00 | 3.3E+00 | 8.9E-03 | nxrAB | pre |
| RZ - YK | -1.5E+00 | 5.1E-01 | 23 | -3.0E+00 | -6.8E-02 | -3.0E+00 | 1.2E-02 | hzo | pre |
| eLL - YK | -1.8E+00 | 5.5E-01 | 23 | -3.4E+00 | -2.2E-01 | -3.3E+00 | 1.1E-02 | hzo | pre |
| LL - YK | -1.7E+00 | 5.1E-01 | 23 | -3.1E+00 | -1.9E-01 | -3.3E+00 | 1.1E-02 | hzo | pre |
| RZ - YK | 1.5E+00 | 4.5E-01 | 23 | 2.3E-01 | 2.9E+00 | 3.4E+00 | 1.5E-02 | hzsABC | pre |
| RZ - YK | 1.1E+00 | 4.2E-01 | 23 | -1.4E-01 | 2.3E+00 | 2.6E+00 | 3.6E-02 | napABC | pre |
| eLL - YK | 1.2E+00 | 4.5E-01 | 23 | -5.0E-02 | 2.5E+00 | 2.8E+00 | 3.2E-02 | napABC | pre |
| LL - YK | 1.4E+00 | 4.2E-01 | 23 | 1.9E-01 | 2.6E+00 | 3.3E+00 | 1.7E-02 | napABC | pre |
| RZ - LL | -1.3E+00 | 3.4E-01 | 23 | -2.2E+00 | -2.8E-01 | -3.7E+00 | 1.7E-03 | narGHJI | pre |
| RZ - YK | -2.7E+00 | 3.4E-01 | 23 | -3.7E+00 | -1.7E+00 | -7.9E+00 | 2.9E-07 | narGHJI | pre |
| eLL - YK | -2.1E+00 | 3.7E-01 | 23 | -3.2E+00 | -1.1E+00 | -5.8E+00 | 1.9E-05 | narGHJI | pre |
| LL - YK | -1.4E+00 | 3.4E-01 | 23 | -2.4E+00 | -4.6E-01 | -4.2E+00 | 6.2E-04 | narGHJI | pre |
| RZ - LL | -1.2E+00 | 4.6E-01 | 23 | -2.5E+00 | 1.2E-01 | -2.6E+00 | 2.3E-02 | narVWYZ | pre |
| RZ - YK | -2.4E+00 | 4.6E-01 | 23 | -3.8E+00 | -1.1E+00 | -5.3E+00 | 1.4E-04 | narVWYZ | pre |
| eLL - YK | -2.2E+00 | 5.0E-01 | 23 | -3.6E+00 | -7.1E-01 | -4.3E+00 | 7.7E-04 | narVWYZ | pre |
| LL - YK | -1.2E+00 | 4.6E-01 | 23 | -2.6E+00 | 1.1E-01 | -2.6E+00 | 2.3E-02 | narVWYZ | pre |
| RZ - YK | 2.2E+00 | 2.3E-01 | 23 | 1.5E+00 | 2.9E+00 | 9.3E+00 | 8.4E-09 | nirK | pre |
| eLL - LL | 5.8E-01 | 2.5E-01 | 23 | -1.5E-01 | 1.3E+00 | 2.3E+00 | 4.9E-02 | nirK | pre |
| eLL - YK | 2.4E+00 | 2.5E-01 | 23 | 1.7E+00 | 3.2E+00 | 9.6E+00 | 8.4E-09 | nirK | pre |
| LL - YK | 1.8E+00 | 2.3E-01 | 23 | 1.2E+00 | 2.5E+00 | 7.9E+00 | 1.1E-07 | nirK | pre |
| RZ - YK | 2.1E+00 | 3.3E-01 | 23 | 1.1E+00 | 3.0E+00 | 6.2E+00 | 1.6E-05 | nirS | pre |
| eLL - YK | 1.9E+00 | 3.6E-01 | 23 | 9.0E-01 | 3.0E+00 | 5.4E+00 | 5.4E-05 | nirS | pre |
| LL - YK | 1.3E+00 | 3.3E-01 | 23 | 3.8E-01 | 2.3E+00 | 4.0E+00 | 1.0E-03 | nirS | pre |
| RZ - eLL | -1.3E+00 | 3.8E-01 | 23 | -2.4E+00 | -2.3E-01 | -3.5E+00 | 4.0E-03 | norBC | pre |
| RZ - LL | -2.1E+00 | 3.5E-01 | 23 | -3.1E+00 | -1.1E+00 | -6.1E+00 | 2.0E-05 | norBC | pre |
| RZ - YK | -1.6E+00 | 3.5E-01 | 23 | -2.7E+00 | -6.4E-01 | -4.7E+00 | 2.8E-04 | norBC | pre |
| RZ - eLL | -1.6E+00 | 5.8E-01 | 23 | -3.2E+00 | 8.1E-02 | -2.7E+00 | 2.2E-02 | nosZ | pre |
| RZ - LL | -1.4E+00 | 5.3E-01 | 23 | -2.9E+00 | 1.3E-01 | -2.6E+00 | 2.2E-02 | nosZ | pre |
| eLL - YK | 1.8E+00 | 5.8E-01 | 23 | 1.0E-01 | 3.4E+00 | 3.1E+00 | 2.0E-02 | nosZ | pre |
| LL - YK | 1.6E+00 | 5.3E-01 | 23 | 5.4E-02 | 3.1E+00 | 3.0E+00 | 2.0E-02 | nosZ | pre |
| RZ - LL | 7.7E-01 | 3.4E-01 | 23 | -2.1E-01 | 1.7E+00 | 2.3E+00 | 4.8E-02 | nrfABCD | pre |
| RZ - YK | 2.4E+00 | 3.4E-01 | 23 | 1.4E+00 | 3.3E+00 | 7.0E+00 | 2.2E-06 | nrfABCD | pre |
| eLL - YK | 1.8E+00 | 3.6E-01 | 23 | 7.9E-01 | 2.9E+00 | 5.0E+00 | 1.3E-04 | nrfABCD | pre |
| LL - YK | 1.6E+00 | 3.4E-01 | 23 | 6.3E-01 | 2.6E+00 | 4.7E+00 | 1.8E-04 | nrfABCD | pre |
| RZ - eLL | 9.5E-01 | 3.0E-01 | 23 | 7.3E-02 | 1.8E+00 | 3.1E+00 | 8.7E-03 | nifDHKWanfG | pre |
| RZ - LL | 1.9E+00 | 2.8E-01 | 23 | 1.1E+00 | 2.7E+00 | 6.7E+00 | 4.2E-06 | nifDHKWanfG | pre |
| RZ - YK | 1.8E+00 | 2.8E-01 | 23 | 1.0E+00 | 2.6E+00 | 6.4E+00 | 4.2E-06 | nifDHKWanfG | pre |
| eLL - LL | 9.2E-01 | 3.0E-01 | 23 | 4.7E-02 | 1.8E+00 | 3.0E+00 | 8.7E-03 | nifDHKWanfG | pre |
| eLL - YK | 8.6E-01 | 3.0E-01 | 23 | -1.4E-02 | 1.7E+00 | 2.8E+00 | 1.1E-02 | nifDHKWanfG | pre |
| RZ - eLL | 7.2E-01 | 3.0E-01 | 23 | -1.4E-01 | 1.6E+00 | 2.4E+00 | 2.9E-02 | nasAB | pre |
| RZ - LL | 1.0E+00 | 2.7E-01 | 23 | 2.4E-01 | 1.8E+00 | 3.8E+00 | 1.6E-03 | nasAB | pre |
| RZ - YK | 2.7E+00 | 2.7E-01 | 23 | 1.9E+00 | 3.5E+00 | 9.8E+00 | 7.2E-09 | nasAB | pre |
| eLL - YK | 2.0E+00 | 3.0E-01 | 23 | 1.1E+00 | 2.8E+00 | 6.6E+00 | 2.8E-06 | nasAB | pre |
| LL - YK | 1.7E+00 | 2.7E-01 | 23 | 8.6E-01 | 2.4E+00 | 6.0E+00 | 7.9E-06 | nasAB | pre |
| RZ - eLL | -1.0E+00 | 1.8E-01 | 23 | -1.5E+00 | -5.3E-01 | -5.9E+00 | 8.1E-06 | narAB | pre |
| RZ - LL | -1.8E+00 | 1.6E-01 | 23 | -2.2E+00 | -1.3E+00 | -1.1E+01 | 5.6E-10 | narAB | pre |
| RZ - YK | -2.6E+00 | 1.6E-01 | 23 | -3.0E+00 | -2.1E+00 | -1.6E+01 | 5.9E-13 | narAB | pre |
| eLL - LL | -7.2E-01 | 1.8E-01 | 23 | -1.2E+00 | -2.1E-01 | -4.1E+00 | 4.5E-04 | narAB | pre |
| eLL - YK | -1.5E+00 | 1.8E-01 | 23 | -2.0E+00 | -1.0E+00 | -8.6E+00 | 2.6E-08 | narAB | pre |
| LL - YK | -7.9E-01 | 1.6E-01 | 23 | -1.3E+00 | -3.2E-01 | -4.8E+00 | 8.2E-05 | narAB | pre |
| RZ - LL | 1.4E+00 | 4.3E-01 | 23 | 1.0E-01 | 2.6E+00 | 3.1E+00 | 2.9E-02 | ureABC | pre |
| RZ - LL | -1.1E+00 | 3.5E-01 | 23 | -2.1E+00 | -6.3E-02 | -3.1E+00 | 8.2E-03 | nao | pre |
| RZ - YK | -2.6E+00 | 3.5E-01 | 23 | -3.6E+00 | -1.6E+00 | -7.4E+00 | 1.0E-06 | nao | pre |
| eLL - YK | -2.3E+00 | 3.8E-01 | 23 | -3.4E+00 | -1.2E+00 | -6.0E+00 | 1.2E-05 | nao | pre |
| LL - YK | -1.5E+00 | 3.5E-01 | 23 | -2.5E+00 | -4.9E-01 | -4.3E+00 | 5.4E-04 | nao | pre |
| RZ - YK | -2.2E+00 | 3.0E-01 | 23 | -3.1E+00 | -1.3E+00 | -7.2E+00 | 1.4E-06 | nmo | pre |
| eLL - YK | -1.8E+00 | 3.3E-01 | 23 | -2.8E+00 | -8.6E-01 | -5.5E+00 | 2.8E-05 | nmo | pre |
| LL - YK | -1.8E+00 | 3.0E-01 | 23 | -2.7E+00 | -9.6E-01 | -6.0E+00 | 1.1E-05 | nmo | pre |
| RZ - eLL | 1.1E+00 | 3.0E-01 | 23 | 2.7E-01 | 2.0E+00 | 3.8E+00 | 1.9E-03 | gdh | pre |
| RZ - LL | 1.7E+00 | 2.8E-01 | 23 | 8.4E-01 | 2.5E+00 | 5.9E+00 | 1.5E-05 | gdh | pre |
| RZ - YK | 1.9E+00 | 2.8E-01 | 23 | 1.1E+00 | 2.7E+00 | 6.7E+00 | 4.6E-06 | gdh | pre |
| eLL - YK | 7.3E-01 | 3.0E-01 | 23 | -1.4E-01 | 1.6E+00 | 2.4E+00 | 3.5E-02 | gdh | pre |
| RZ - eLL | -2.0E+00 | 5.0E-01 | 23 | -3.5E+00 | -5.7E-01 | -4.0E+00 | 1.6E-03 | ans | pre |
| RZ - LL | -2.3E+00 | 4.6E-01 | 23 | -3.6E+00 | -9.7E-01 | -5.0E+00 | 2.9E-04 | ans | pre |
| RZ - YK | -1.7E+00 | 4.6E-01 | 23 | -3.1E+00 | -4.0E-01 | -3.8E+00 | 2.1E-03 | ans | pre |
| RZ - YK | -2.0E+00 | 1.6E-01 | 23 | -2.5E+00 | -1.6E+00 | -1.3E+01 | 3.8E-11 | gs | pre |
| eLL - YK | -1.9E+00 | 1.7E-01 | 23 | -2.4E+00 | -1.4E+00 | -1.1E+01 | 1.7E-10 | gs | pre |
| LL - YK | -1.9E+00 | 1.6E-01 | 23 | -2.3E+00 | -1.4E+00 | -1.2E+01 | 1.2E-10 | gs | pre |
| RZ - eLL | 8.1E-01 | 3.7E-01 | 23 | -2.5E-01 | 1.9E+00 | 2.2E+00 | 4.6E-02 | gln | pre |
| RZ - LL | 1.3E+00 | 3.4E-01 | 23 | 2.6E-01 | 2.2E+00 | 3.7E+00 | 2.6E-03 | gln | pre |
| RZ - YK | 2.5E+00 | 3.4E-01 | 23 | 1.5E+00 | 3.4E+00 | 7.2E+00 | 1.6E-06 | gln | pre |
| eLL - YK | 1.6E+00 | 3.7E-01 | 23 | 5.8E-01 | 2.7E+00 | 4.5E+00 | 5.5E-04 | gln | pre |
| LL - YK | 1.2E+00 | 3.4E-01 | 23 | 2.2E-01 | 2.2E+00 | 3.5E+00 | 2.7E-03 | gln | pre |
| RZ - YK | 2.1E+00 | 4.9E-01 | 23 | 6.6E-01 | 3.5E+00 | 4.2E+00 | 1.8E-03 | pmoABC | pre |
| RZ - eLL | 1.4E+00 | 3.8E-01 | 23 | 3.2E-01 | 2.5E+00 | 3.7E+00 | 1.7E-03 | hcp | pre |
| RZ - LL | 1.7E+00 | 3.5E-01 | 23 | 6.9E-01 | 2.7E+00 | 4.8E+00 | 2.1E-04 | hcp | pre |
| eLL - YK | -1.5E+00 | 3.8E-01 | 23 | -2.6E+00 | -4.1E-01 | -4.0E+00 | 1.2E-03 | hcp | pre |
| LL - YK | -1.8E+00 | 3.5E-01 | 23 | -2.8E+00 | -7.9E-01 | -5.1E+00 | 2.1E-04 | hcp | pre |
| RZ - eLL | -7.2E-01 | 1.6E-01 | 23 | -1.2E+00 | -2.6E-01 | -4.5E+00 | 2.2E-04 | amoABC_AOA | post |
| RZ - LL | -5.7E-01 | 1.6E-01 | 23 | -1.0E+00 | -1.1E-01 | -3.6E+00 | 1.9E-03 | amoABC_AOA | post |
| RZ - YK | 1.7E+00 | 1.6E-01 | 23 | 1.3E+00 | 2.2E+00 | 1.1E+01 | 2.9E-10 | amoABC_AOA | post |
| eLL - YK | 2.5E+00 | 1.6E-01 | 23 | 2.0E+00 | 2.9E+00 | 1.5E+01 | 7.3E-13 | amoABC_AOA | post |
| LL - YK | 2.3E+00 | 1.6E-01 | 23 | 1.8E+00 | 2.8E+00 | 1.4E+01 | 1.4E-12 | amoABC_AOA | post |
| RZ - LL | 1.5E+00 | 4.8E-01 | 23 | 6.8E-02 | 2.8E+00 | 3.0E+00 | 1.8E-02 | amoABC_AOB | post |
| LL - YK | -1.7E+00 | 4.8E-01 | 23 | -3.1E+00 | -3.4E-01 | -3.6E+00 | 9.1E-03 | amoABC_AOB | post |
| RZ - eLL | -1.7E+00 | 5.4E-01 | 23 | -3.3E+00 | -1.9E-01 | -3.2E+00 | 1.1E-02 | hao | post |
| RZ - YK | -2.0E+00 | 5.4E-01 | 23 | -3.5E+00 | -4.3E-01 | -3.7E+00 | 7.3E-03 | hao | post |
| RZ - YK | 1.9E+00 | 4.4E-01 | 23 | 6.3E-01 | 3.2E+00 | 4.3E+00 | 1.5E-03 | nxrAB | post |
| eLL - YK | 1.5E+00 | 4.4E-01 | 23 | 2.3E-01 | 2.8E+00 | 3.4E+00 | 7.1E-03 | nxrAB | post |
| LL - YK | 1.2E+00 | 4.4E-01 | 23 | -9.4E-02 | 2.4E+00 | 2.7E+00 | 2.7E-02 | nxrAB | post |
| RZ - LL | 1.7E+00 | 5.1E-01 | 23 | 2.6E-01 | 3.2E+00 | 3.4E+00 | 1.5E-02 | hzo | post |
| LL - YK | -1.5E+00 | 5.1E-01 | 23 | -3.0E+00 | -2.2E-02 | -2.9E+00 | 2.3E-02 | hzo | post |
| eLL - YK | 1.8E+00 | 4.5E-01 | 23 | 5.3E-01 | 3.2E+00 | 4.1E+00 | 2.9E-03 | hzsABC | post |
| RZ - YK | 1.6E+00 | 4.2E-01 | 23 | 3.6E-01 | 2.8E+00 | 3.8E+00 | 2.1E-03 | napABC | post |
| eLL - YK | 2.3E+00 | 4.2E-01 | 23 | 1.1E+00 | 3.5E+00 | 5.6E+00 | 3.3E-05 | napABC | post |
| LL - YK | 2.3E+00 | 4.2E-01 | 23 | 1.1E+00 | 3.5E+00 | 5.6E+00 | 3.3E-05 | napABC | post |
| RZ - LL | -9.1E-01 | 3.4E-01 | 23 | -1.9E+00 | 7.1E-02 | -2.7E+00 | 2.0E-02 | narGHJI | post |
| RZ - YK | -2.0E+00 | 3.4E-01 | 23 | -2.9E+00 | -9.7E-01 | -5.7E+00 | 4.6E-05 | narGHJI | post |
| eLL - YK | -1.4E+00 | 3.4E-01 | 23 | -2.4E+00 | -4.3E-01 | -4.2E+00 | 1.1E-03 | narGHJI | post |
| LL - YK | -1.0E+00 | 3.4E-01 | 23 | -2.0E+00 | -5.9E-02 | -3.1E+00 | 1.1E-02 | narGHJI | post |
| RZ - YK | -1.5E+00 | 4.6E-01 | 23 | -2.9E+00 | -2.0E-01 | -3.3E+00 | 1.8E-02 | narVWYZ | post |
| eLL - YK | -1.4E+00 | 4.6E-01 | 23 | -2.7E+00 | -2.6E-02 | -2.9E+00 | 2.2E-02 | narVWYZ | post |
| RZ - eLL | -6.7E-01 | 2.3E-01 | 23 | -1.3E+00 | 2.7E-03 | -2.9E+00 | 1.3E-02 | nirK | post |
| RZ - LL | -5.9E-01 | 2.3E-01 | 23 | -1.3E+00 | 8.6E-02 | -2.5E+00 | 2.3E-02 | nirK | post |
| RZ - YK | 1.6E+00 | 2.3E-01 | 23 | 9.4E-01 | 2.3E+00 | 6.9E+00 | 9.6E-07 | nirK | post |
| eLL - YK | 2.3E+00 | 2.3E-01 | 23 | 1.6E+00 | 3.0E+00 | 9.8E+00 | 6.9E-09 | nirK | post |
| LL - YK | 2.2E+00 | 2.3E-01 | 23 | 1.5E+00 | 2.9E+00 | 9.4E+00 | 6.9E-09 | nirK | post |
| RZ - YK | 2.2E+00 | 3.3E-01 | 23 | 1.3E+00 | 3.2E+00 | 6.6E+00 | 2.7E-06 | nirS | post |
| eLL - YK | 2.4E+00 | 3.3E-01 | 23 | 1.4E+00 | 3.4E+00 | 7.2E+00 | 1.5E-06 | nirS | post |
| LL - YK | 1.7E+00 | 3.3E-01 | 23 | 6.9E-01 | 2.6E+00 | 5.0E+00 | 1.0E-04 | nirS | post |
| RZ - eLL | -1.8E+00 | 3.5E-01 | 23 | -2.8E+00 | -7.6E-01 | -5.1E+00 | 7.8E-05 | norBC | post |
| RZ - LL | -2.4E+00 | 3.5E-01 | 23 | -3.4E+00 | -1.4E+00 | -7.0E+00 | 2.4E-06 | norBC | post |
| RZ - YK | -2.2E+00 | 3.5E-01 | 23 | -3.2E+00 | -1.2E+00 | -6.2E+00 | 6.9E-06 | norBC | post |
| eLL - YK | 1.8E+00 | 5.3E-01 | 23 | 2.8E-01 | 3.4E+00 | 3.4E+00 | 1.4E-02 | nosZ | post |
| LL - YK | 1.4E+00 | 5.3E-01 | 23 | -1.3E-01 | 2.9E+00 | 2.6E+00 | 4.4E-02 | nosZ | post |
| RZ - LL | 1.8E+00 | 5.1E-01 | 23 | 3.2E-01 | 3.2E+00 | 3.5E+00 | 5.5E-03 | NirBD | post |
| LL - YK | -1.8E+00 | 5.1E-01 | 23 | -3.3E+00 | -3.5E-01 | -3.6E+00 | 5.5E-03 | NirBD | post |
| RZ - LL | 8.2E-01 | 3.4E-01 | 23 | -1.6E-01 | 1.8E+00 | 2.4E+00 | 3.6E-02 | nrfABCD | post |
| RZ - YK | 2.4E+00 | 3.4E-01 | 23 | 1.4E+00 | 3.3E+00 | 7.0E+00 | 2.4E-06 | nrfABCD | post |
| eLL - YK | 1.8E+00 | 3.4E-01 | 23 | 8.6E-01 | 2.8E+00 | 5.4E+00 | 4.8E-05 | nrfABCD | post |
| LL - YK | 1.5E+00 | 3.4E-01 | 23 | 5.7E-01 | 2.5E+00 | 4.6E+00 | 2.7E-04 | nrfABCD | post |
| RZ - eLL | 2.0E+00 | 2.8E-01 | 23 | 1.2E+00 | 2.8E+00 | 7.2E+00 | 8.2E-07 | nifDHKWanfG | post |
| RZ - LL | 2.8E+00 | 2.8E-01 | 23 | 2.0E+00 | 3.6E+00 | 9.9E+00 | 5.2E-09 | nifDHKWanfG | post |
| RZ - YK | 1.5E+00 | 2.8E-01 | 23 | 7.4E-01 | 2.4E+00 | 5.5E+00 | 2.6E-05 | nifDHKWanfG | post |
| eLL - LL | 7.8E-01 | 2.8E-01 | 23 | -3.2E-02 | 1.6E+00 | 2.8E+00 | 1.3E-02 | nifDHKWanfG | post |
| LL - YK | -1.2E+00 | 2.8E-01 | 23 | -2.0E+00 | -4.3E-01 | -4.4E+00 | 3.0E-04 | nifDHKWanfG | post |
| RZ - LL | 1.1E+00 | 2.7E-01 | 23 | 3.4E-01 | 1.9E+00 | 4.1E+00 | 8.2E-04 | nasAB | post |
| RZ - YK | 1.9E+00 | 2.7E-01 | 23 | 1.2E+00 | 2.7E+00 | 7.1E+00 | 1.9E-06 | nasAB | post |
| eLL - LL | 6.5E-01 | 2.7E-01 | 23 | -1.5E-01 | 1.4E+00 | 2.4E+00 | 3.3E-02 | nasAB | post |
| eLL - YK | 1.5E+00 | 2.7E-01 | 23 | 6.7E-01 | 2.3E+00 | 5.3E+00 | 6.4E-05 | nasAB | post |
| LL - YK | 8.1E-01 | 2.7E-01 | 23 | 2.1E-02 | 1.6E+00 | 3.0E+00 | 1.0E-02 | nasAB | post |
| RZ - LL | -1.1E+00 | 4.8E-01 | 23 | -2.5E+00 | 2.4E-01 | -2.4E+00 | 3.9E-02 | NR | post |
| RZ - YK | 1.2E+00 | 4.8E-01 | 23 | -1.8E-01 | 2.6E+00 | 2.5E+00 | 3.9E-02 | NR | post |
| eLL - YK | 1.8E+00 | 4.8E-01 | 23 | 4.6E-01 | 3.2E+00 | 3.8E+00 | 2.5E-03 | NR | post |
| LL - YK | 2.3E+00 | 4.8E-01 | 23 | 9.6E-01 | 3.7E+00 | 4.9E+00 | 3.7E-04 | NR | post |
| RZ - eLL | -1.4E+00 | 1.6E-01 | 23 | -1.8E+00 | -8.9E-01 | -8.3E+00 | 4.4E-08 | narAB | post |
| RZ - LL | -1.7E+00 | 1.6E-01 | 23 | -2.2E+00 | -1.2E+00 | -1.0E+01 | 1.1E-09 | narAB | post |
| RZ - YK | -2.7E+00 | 1.6E-01 | 23 | -3.1E+00 | -2.2E+00 | -1.6E+01 | 2.4E-13 | narAB | post |
| eLL - LL | -3.4E-01 | 1.6E-01 | 23 | -8.2E-01 | 1.3E-01 | -2.1E+00 | 4.7E-02 | narAB | post |
| eLL - YK | -1.3E+00 | 1.6E-01 | 23 | -1.8E+00 | -8.3E-01 | -8.0E+00 | 6.6E-08 | narAB | post |
| LL - YK | -9.6E-01 | 1.6E-01 | 23 | -1.4E+00 | -4.9E-01 | -5.9E+00 | 6.4E-06 | narAB | post |
| RZ - LL | 2.1E+00 | 4.3E-01 | 23 | 8.4E-01 | 3.4E+00 | 4.8E+00 | 4.3E-04 | ureABC | post |
| eLL - LL | 1.7E+00 | 4.3E-01 | 23 | 4.0E-01 | 2.9E+00 | 3.8E+00 | 2.6E-03 | ureABC | post |
| LL - YK | -1.6E+00 | 4.3E-01 | 23 | -2.8E+00 | -3.4E-01 | -3.7E+00 | 2.6E-03 | ureABC | post |
| RZ - LL | -1.0E+00 | 3.5E-01 | 23 | -2.0E+00 | -1.6E-02 | -2.9E+00 | 1.5E-02 | nao | post |
| RZ - YK | -2.0E+00 | 3.5E-01 | 23 | -3.0E+00 | -9.5E-01 | -5.6E+00 | 6.3E-05 | nao | post |
| eLL - YK | -1.4E+00 | 3.5E-01 | 23 | -2.4E+00 | -4.1E-01 | -4.1E+00 | 1.5E-03 | nao | post |
| LL - YK | -9.4E-01 | 3.5E-01 | 23 | -1.9E+00 | 7.4E-02 | -2.7E+00 | 2.0E-02 | nao | post |
| RZ - YK | -2.4E+00 | 3.0E-01 | 23 | -3.2E+00 | -1.5E+00 | -7.7E+00 | 4.7E-07 | nmo | post |
| eLL - YK | -2.1E+00 | 3.0E-01 | 23 | -3.0E+00 | -1.2E+00 | -6.9E+00 | 1.5E-06 | nmo | post |
| LL - YK | -2.0E+00 | 3.0E-01 | 23 | -2.8E+00 | -1.1E+00 | -6.5E+00 | 2.8E-06 | nmo | post |
| RZ - eLL | 2.3E+00 | 2.8E-01 | 23 | 1.5E+00 | 3.1E+00 | 8.3E+00 | 4.9E-08 | gdh | post |
| RZ - LL | 2.4E+00 | 2.8E-01 | 23 | 1.6E+00 | 3.2E+00 | 8.6E+00 | 3.7E-08 | gdh | post |
| RZ - YK | 2.5E+00 | 2.8E-01 | 23 | 1.6E+00 | 3.3E+00 | 8.8E+00 | 3.7E-08 | gdh | post |
| RZ - LL | -1.8E+00 | 5.6E-01 | 23 | -3.4E+00 | -1.7E-01 | -3.2E+00 | 2.4E-02 | gls | post |
| RZ - YK | -1.5E+00 | 5.6E-01 | 23 | -3.1E+00 | 1.1E-01 | -2.7E+00 | 4.0E-02 | gls | post |
| RZ - eLL | -1.7E+00 | 4.6E-01 | 23 | -3.0E+00 | -3.6E-01 | -3.7E+00 | 5.9E-03 | ans | post |
| RZ - LL | -1.6E+00 | 4.6E-01 | 23 | -2.9E+00 | -2.8E-01 | -3.5E+00 | 5.9E-03 | ans | post |
| RZ - YK | -1.4E+00 | 4.6E-01 | 23 | -2.8E+00 | -1.0E-01 | -3.1E+00 | 9.8E-03 | ans | post |
| RZ - YK | 1.5E+00 | 5.6E-01 | 23 | -8.0E-02 | 3.1E+00 | 2.7E+00 | 2.3E-02 | asn | post |
| eLL - YK | 1.9E+00 | 5.6E-01 | 23 | 3.4E-01 | 3.6E+00 | 3.5E+00 | 8.1E-03 | asn | post |
| LL - YK | 1.9E+00 | 5.6E-01 | 23 | 2.7E-01 | 3.5E+00 | 3.4E+00 | 8.1E-03 | asn | post |
| RZ - YK | -2.4E+00 | 1.6E-01 | 23 | -2.8E+00 | -1.9E+00 | -1.5E+01 | 5.0E-13 | gs | post |
| eLL - YK | -2.4E+00 | 1.6E-01 | 23 | -2.9E+00 | -2.0E+00 | -1.5E+01 | 5.0E-13 | gs | post |
| LL - YK | -2.4E+00 | 1.6E-01 | 23 | -2.9E+00 | -1.9E+00 | -1.5E+01 | 5.0E-13 | gs | post |
| RZ - YK | 2.3E+00 | 3.4E-01 | 23 | 1.3E+00 | 3.3E+00 | 6.7E+00 | 5.2E-06 | gln | post |
| eLL - YK | 1.7E+00 | 3.4E-01 | 23 | 7.2E-01 | 2.7E+00 | 5.0E+00 | 9.3E-05 | gln | post |
| LL - YK | 1.8E+00 | 3.4E-01 | 23 | 8.5E-01 | 2.8E+00 | 5.4E+00 | 5.5E-05 | gln | post |
| eLL - YK | -1.2E+00 | 3.5E-01 | 23 | -2.2E+00 | -1.9E-01 | -3.4E+00 | 7.1E-03 | hcp | post |
| LL - YK | -1.5E+00 | 3.5E-01 | 23 | -2.5E+00 | -4.4E-01 | -4.1E+00 | 2.5E-03 | hcp | post |
| RZ - eLL | -5.5E-01 | 1.5E-01 | 27 | -9.7E-01 | -1.3E-01 | -3.7E+00 | 1.4E-03 | amoABC_AOA | average |
| RZ - YK | 1.9E+00 | 1.4E-01 | 27 | 1.5E+00 | 2.3E+00 | 1.3E+01 | 4.7E-13 | amoABC_AOA | average |
| eLL - LL | 3.9E-01 | 1.5E-01 | 27 | -2.6E-02 | 8.2E-01 | 2.7E+00 | 1.5E-02 | amoABC_AOA | average |
| eLL - YK | 2.4E+00 | 1.5E-01 | 27 | 2.0E+00 | 2.9E+00 | 1.7E+01 | 7.0E-15 | amoABC_AOA | average |
| LL - YK | 2.1E+00 | 1.4E-01 | 27 | 1.6E+00 | 2.5E+00 | 1.4E+01 | 1.1E-13 | amoABC_AOA | average |
| RZ - YK | -1.0E+00 | 3.9E-01 | 27 | -2.1E+00 | 9.1E-02 | -2.6E+00 | 2.9E-02 | amoABC_AOB | average |
| eLL - YK | -1.4E+00 | 4.1E-01 | 27 | -2.6E+00 | -2.9E-01 | -3.6E+00 | 4.3E-03 | amoABC_AOB | average |
| LL - YK | -1.7E+00 | 3.9E-01 | 27 | -2.8E+00 | -5.8E-01 | -4.3E+00 | 1.2E-03 | amoABC_AOB | average |
| RZ - YK | -1.7E+00 | 4.0E-01 | 27 | -2.9E+00 | -6.0E-01 | -4.3E+00 | 1.1E-03 | hao | average |
| RZ - LL | 1.3E+00 | 4.0E-01 | 27 | 1.9E-01 | 2.5E+00 | 3.3E+00 | 7.7E-03 | nxrAB | average |
| RZ - YK | 1.7E+00 | 4.0E-01 | 27 | 5.3E-01 | 2.8E+00 | 4.2E+00 | 1.7E-03 | nxrAB | average |
| eLL - YK | -1.3E+00 | 4.3E-01 | 27 | -2.5E+00 | -4.0E-02 | -2.9E+00 | 2.0E-02 | hzo | average |
| LL - YK | -1.6E+00 | 4.1E-01 | 27 | -2.8E+00 | -4.1E-01 | -3.8E+00 | 4.1E-03 | hzo | average |
| RZ - YK | 1.2E+00 | 4.3E-01 | 27 | -4.4E-02 | 2.4E+00 | 2.7E+00 | 3.2E-02 | hzsABC | average |
| eLL - YK | 1.6E+00 | 4.4E-01 | 27 | 3.1E-01 | 2.8E+00 | 3.5E+00 | 8.7E-03 | hzsABC | average |
| RZ - YK | 1.3E+00 | 3.4E-01 | 27 | 3.4E-01 | 2.3E+00 | 3.8E+00 | 1.4E-03 | napABC | average |
| eLL - YK | 1.8E+00 | 3.5E-01 | 27 | 7.6E-01 | 2.8E+00 | 5.0E+00 | 9.1E-05 | napABC | average |
| LL - YK | 1.9E+00 | 3.4E-01 | 27 | 8.8E-01 | 2.8E+00 | 5.4E+00 | 5.9E-05 | napABC | average |
| RZ - eLL | -5.6E-01 | 2.5E-01 | 27 | -1.3E+00 | 1.6E-01 | -2.2E+00 | 4.2E-02 | narGHJI | average |
| RZ - LL | -1.1E+00 | 2.4E-01 | 27 | -1.8E+00 | -4.0E-01 | -4.5E+00 | 1.8E-04 | narGHJI | average |
| RZ - YK | -2.3E+00 | 2.4E-01 | 27 | -3.0E+00 | -1.6E+00 | -9.6E+00 | 2.0E-09 | narGHJI | average |
| eLL - LL | -5.3E-01 | 2.5E-01 | 27 | -1.2E+00 | 1.8E-01 | -2.1E+00 | 4.4E-02 | narGHJI | average |
| eLL - YK | -1.8E+00 | 2.5E-01 | 27 | -2.5E+00 | -1.1E+00 | -7.1E+00 | 4.1E-07 | narGHJI | average |
| LL - YK | -1.2E+00 | 2.4E-01 | 27 | -1.9E+00 | -5.5E-01 | -5.1E+00 | 4.4E-05 | narGHJI | average |
| RZ - LL | -9.7E-01 | 3.2E-01 | 27 | -1.9E+00 | -5.7E-02 | -3.0E+00 | 8.1E-03 | narVWYZ | average |
| RZ - YK | -2.0E+00 | 3.2E-01 | 27 | -2.9E+00 | -1.1E+00 | -6.2E+00 | 7.6E-06 | narVWYZ | average |
| eLL - LL | -7.4E-01 | 3.3E-01 | 27 | -1.7E+00 | 2.1E-01 | -2.2E+00 | 4.3E-02 | narVWYZ | average |
| eLL - YK | -1.8E+00 | 3.3E-01 | 27 | -2.7E+00 | -8.1E-01 | -5.3E+00 | 4.3E-05 | narVWYZ | average |
| LL - YK | -1.0E+00 | 3.2E-01 | 27 | -1.9E+00 | -1.0E-01 | -3.2E+00 | 7.5E-03 | narVWYZ | average |
| RZ - eLL | -4.4E-01 | 1.9E-01 | 27 | -9.8E-01 | 9.4E-02 | -2.3E+00 | 4.0E-02 | nirK | average |
| RZ - YK | 1.9E+00 | 1.8E-01 | 27 | 1.4E+00 | 2.4E+00 | 1.0E+01 | 1.0E-10 | nirK | average |
| eLL - YK | 2.3E+00 | 1.9E-01 | 27 | 1.8E+00 | 2.9E+00 | 1.2E+01 | 6.2E-12 | nirK | average |
| LL - YK | 2.0E+00 | 1.8E-01 | 27 | 1.5E+00 | 2.5E+00 | 1.1E+01 | 3.8E-11 | nirK | average |
| RZ - LL | 6.4E-01 | 2.3E-01 | 27 | -2.6E-02 | 1.3E+00 | 2.7E+00 | 1.3E-02 | nirS | average |
| RZ - YK | 2.1E+00 | 2.3E-01 | 27 | 1.5E+00 | 2.8E+00 | 9.2E+00 | 3.8E-09 | nirS | average |
| eLL - LL | 6.7E-01 | 2.4E-01 | 27 | -1.8E-02 | 1.4E+00 | 2.8E+00 | 1.3E-02 | nirS | average |
| eLL - YK | 2.2E+00 | 2.4E-01 | 27 | 1.5E+00 | 2.9E+00 | 9.0E+00 | 3.8E-09 | nirS | average |
| LL - YK | 1.5E+00 | 2.3E-01 | 27 | 8.4E-01 | 2.2E+00 | 6.5E+00 | 1.3E-06 | nirS | average |
| RZ - eLL | -1.5E+00 | 2.4E-01 | 27 | -2.2E+00 | -8.6E-01 | -6.4E+00 | 1.5E-06 | norBC | average |
| RZ - LL | -2.3E+00 | 2.3E-01 | 27 | -2.9E+00 | -1.6E+00 | -9.7E+00 | 1.5E-09 | norBC | average |
| RZ - YK | -1.9E+00 | 2.3E-01 | 27 | -2.6E+00 | -1.2E+00 | -8.2E+00 | 2.7E-08 | norBC | average |
| eLL - LL | -7.3E-01 | 2.4E-01 | 27 | -1.4E+00 | -4.2E-02 | -3.0E+00 | 8.2E-03 | norBC | average |
| RZ - eLL | -1.3E+00 | 3.7E-01 | 27 | -2.4E+00 | -2.7E-01 | -3.6E+00 | 2.7E-03 | nosZ | average |
| RZ - LL | -1.0E+00 | 3.6E-01 | 27 | -2.0E+00 | -1.3E-02 | -2.9E+00 | 1.1E-02 | nosZ | average |
| eLL - YK | 1.8E+00 | 3.7E-01 | 27 | 7.4E-01 | 2.8E+00 | 4.8E+00 | 2.8E-04 | nosZ | average |
| LL - YK | 1.5E+00 | 3.6E-01 | 27 | 4.8E-01 | 2.5E+00 | 4.2E+00 | 7.7E-04 | nosZ | average |
| RZ - eLL | 5.3E-01 | 2.3E-01 | 27 | -1.2E-01 | 1.2E+00 | 2.3E+00 | 3.3E-02 | nrfABCD | average |
| RZ - LL | 7.9E-01 | 2.2E-01 | 27 | 1.6E-01 | 1.4E+00 | 3.6E+00 | 1.9E-03 | nrfABCD | average |
| RZ - YK | 2.4E+00 | 2.2E-01 | 27 | 1.7E+00 | 3.0E+00 | 1.1E+01 | 1.9E-10 | nrfABCD | average |
| eLL - YK | 1.8E+00 | 2.3E-01 | 27 | 1.2E+00 | 2.5E+00 | 8.0E+00 | 3.7E-08 | nrfABCD | average |
| LL - YK | 1.6E+00 | 2.2E-01 | 27 | 9.5E-01 | 2.2E+00 | 7.1E+00 | 2.3E-07 | nrfABCD | average |
| RZ - eLL | 1.5E+00 | 2.6E-01 | 27 | 8.0E-01 | 2.3E+00 | 5.9E+00 | 4.9E-06 | nifDHKWanfG | average |
| RZ - LL | 2.3E+00 | 2.5E-01 | 27 | 1.6E+00 | 3.0E+00 | 9.3E+00 | 4.3E-09 | nifDHKWanfG | average |
| RZ - YK | 1.7E+00 | 2.5E-01 | 27 | 9.6E-01 | 2.4E+00 | 6.7E+00 | 1.1E-06 | nifDHKWanfG | average |
| eLL - LL | 7.8E-01 | 2.6E-01 | 27 | 4.1E-02 | 1.5E+00 | 3.0E+00 | 8.5E-03 | nifDHKWanfG | average |
| LL - YK | -6.5E-01 | 2.5E-01 | 27 | -1.4E+00 | 6.6E-02 | -2.6E+00 | 1.9E-02 | nifDHKWanfG | average |
| RZ - eLL | 5.6E-01 | 2.6E-01 | 27 | -1.8E-01 | 1.3E+00 | 2.2E+00 | 4.7E-02 | nasAB | average |
| RZ - LL | 1.1E+00 | 2.5E-01 | 27 | 3.7E-01 | 1.8E+00 | 4.3E+00 | 2.8E-04 | nasAB | average |
| RZ - YK | 2.3E+00 | 2.5E-01 | 27 | 1.6E+00 | 3.0E+00 | 9.3E+00 | 4.3E-09 | nasAB | average |
| eLL - YK | 1.8E+00 | 2.6E-01 | 27 | 1.0E+00 | 2.5E+00 | 6.8E+00 | 8.4E-07 | nasAB | average |
| LL - YK | 1.2E+00 | 2.5E-01 | 27 | 5.2E-01 | 1.9E+00 | 4.9E+00 | 7.3E-05 | nasAB | average |
| RZ - eLL | 1.6E+00 | 4.3E-01 | 27 | 3.5E-01 | 2.8E+00 | 3.7E+00 | 6.5E-03 | nirA | average |
| RZ - LL | 1.2E+00 | 4.2E-01 | 27 | 3.1E-02 | 2.4E+00 | 2.9E+00 | 1.9E-02 | nirA | average |
| RZ - YK | 1.2E+00 | 4.2E-01 | 27 | -2.7E-02 | 2.4E+00 | 2.8E+00 | 1.9E-02 | nirA | average |
| LL - YK | 1.7E+00 | 4.2E-01 | 27 | 4.6E-01 | 2.8E+00 | 4.0E+00 | 3.0E-03 | NR | average |
| RZ - eLL | -1.2E+00 | 1.2E-01 | 27 | -1.6E+00 | -8.8E-01 | -1.0E+01 | 1.5E-10 | narAB | average |
| RZ - LL | -1.7E+00 | 1.2E-01 | 27 | -2.1E+00 | -1.4E+00 | -1.5E+01 | 4.6E-14 | narAB | average |
| RZ - YK | -2.6E+00 | 1.2E-01 | 27 | -2.9E+00 | -2.3E+00 | -2.2E+01 | 3.3E-18 | narAB | average |
| eLL - LL | -5.1E-01 | 1.2E-01 | 27 | -8.5E-01 | -1.7E-01 | -4.2E+00 | 2.5E-04 | narAB | average |
| eLL - YK | -1.4E+00 | 1.2E-01 | 27 | -1.7E+00 | -1.0E+00 | -1.2E+01 | 1.3E-11 | narAB | average |
| LL - YK | -8.8E-01 | 1.2E-01 | 27 | -1.2E+00 | -5.5E-01 | -7.5E+00 | 4.9E-08 | narAB | average |
| RZ - LL | 1.7E+00 | 4.0E-01 | 27 | 5.9E-01 | 2.9E+00 | 4.3E+00 | 1.1E-03 | ureABC | average |
| eLL - LL | 1.3E+00 | 4.1E-01 | 27 | 1.1E-01 | 2.5E+00 | 3.1E+00 | 1.3E-02 | ureABC | average |
| LL - YK | -1.1E+00 | 4.0E-01 | 27 | -2.3E+00 | -2.2E-03 | -2.9E+00 | 1.6E-02 | ureABC | average |
| RZ - LL | -1.0E+00 | 2.5E-01 | 27 | -1.8E+00 | -3.3E-01 | -4.2E+00 | 4.4E-04 | nao | average |
| RZ - YK | -2.3E+00 | 2.5E-01 | 27 | -3.0E+00 | -1.5E+00 | -9.0E+00 | 8.1E-09 | nao | average |
| eLL - LL | -6.2E-01 | 2.6E-01 | 27 | -1.4E+00 | 1.2E-01 | -2.4E+00 | 3.0E-02 | nao | average |
| eLL - YK | -1.8E+00 | 2.6E-01 | 27 | -2.6E+00 | -1.1E+00 | -7.0E+00 | 4.4E-07 | nao | average |
| LL - YK | -1.2E+00 | 2.5E-01 | 27 | -1.9E+00 | -5.0E-01 | -4.8E+00 | 9.7E-05 | nao | average |
| RZ - YK | -2.3E+00 | 2.0E-01 | 27 | -2.9E+00 | -1.7E+00 | -1.1E+01 | 5.9E-11 | nmo | average |
| eLL - YK | -2.0E+00 | 2.1E-01 | 27 | -2.6E+00 | -1.4E+00 | -9.4E+00 | 1.0E-09 | nmo | average |
| LL - YK | -1.9E+00 | 2.0E-01 | 27 | -2.5E+00 | -1.3E+00 | -9.4E+00 | 1.0E-09 | nmo | average |
| RZ - eLL | 1.8E+00 | 2.4E-01 | 27 | 1.1E+00 | 2.5E+00 | 7.5E+00 | 8.1E-08 | gdh | average |
| RZ - LL | 2.0E+00 | 2.3E-01 | 27 | 1.4E+00 | 2.7E+00 | 8.9E+00 | 5.5E-09 | gdh | average |
| RZ - YK | 2.2E+00 | 2.3E-01 | 27 | 1.5E+00 | 2.8E+00 | 9.5E+00 | 2.8E-09 | gdh | average |
| RZ - LL | -1.6E+00 | 3.9E-01 | 27 | -2.7E+00 | -4.9E-01 | -4.1E+00 | 2.0E-03 | gls | average |
| RZ - YK | -1.4E+00 | 3.9E-01 | 27 | -2.5E+00 | -3.2E-01 | -3.7E+00 | 3.2E-03 | gls | average |
| eLL - LL | -1.1E+00 | 4.0E-01 | 27 | -2.2E+00 | 9.6E-02 | -2.6E+00 | 2.9E-02 | gls | average |
| RZ - eLL | -1.9E+00 | 3.2E-01 | 27 | -2.8E+00 | -9.4E-01 | -5.8E+00 | 1.1E-05 | ans | average |
| RZ - LL | -2.0E+00 | 3.1E-01 | 27 | -2.8E+00 | -1.1E+00 | -6.3E+00 | 5.2E-06 | ans | average |
| RZ - YK | -1.6E+00 | 3.1E-01 | 27 | -2.5E+00 | -7.1E-01 | -5.1E+00 | 4.3E-05 | ans | average |
| RZ - YK | 1.3E+00 | 3.8E-01 | 27 | 2.4E-01 | 2.4E+00 | 3.5E+00 | 3.4E-03 | asn | average |
| eLL - YK | 1.7E+00 | 4.0E-01 | 27 | 5.6E-01 | 2.8E+00 | 4.2E+00 | 1.3E-03 | asn | average |
| LL - YK | 1.5E+00 | 3.8E-01 | 27 | 4.4E-01 | 2.6E+00 | 4.0E+00 | 1.3E-03 | asn | average |
| RZ - YK | -2.2E+00 | 1.4E-01 | 27 | -2.6E+00 | -1.8E+00 | -1.6E+01 | 1.8E-14 | gs | average |
| eLL - YK | -2.2E+00 | 1.4E-01 | 27 | -2.6E+00 | -1.8E+00 | -1.5E+01 | 2.3E-14 | gs | average |
| LL - YK | -2.1E+00 | 1.4E-01 | 27 | -2.5E+00 | -1.7E+00 | -1.5E+01 | 2.1E-14 | gs | average |
| RZ - eLL | 7.0E-01 | 2.5E-01 | 27 | -7.1E-03 | 1.4E+00 | 2.8E+00 | 1.1E-02 | gln | average |
| RZ - LL | 8.4E-01 | 2.4E-01 | 27 | 1.6E-01 | 1.5E+00 | 3.5E+00 | 2.4E-03 | gln | average |
| RZ - YK | 2.4E+00 | 2.4E-01 | 27 | 1.7E+00 | 3.1E+00 | 9.8E+00 | 1.3E-09 | gln | average |
| eLL - YK | 1.7E+00 | 2.5E-01 | 27 | 9.5E-01 | 2.4E+00 | 6.7E+00 | 1.1E-06 | gln | average |
| LL - YK | 1.5E+00 | 2.4E-01 | 27 | 8.4E-01 | 2.2E+00 | 6.3E+00 | 1.8E-06 | gln | average |

^1^contrasts: RZ-eLL, RZ-LL, RZ-YK, eLL-LL, eLL-YK, LL-YK;

Calcaric Regosol (RZ), strongly eroded Nudiargic Luvisol (eLL), non-eroded Calcic Luvisol (LL) and colluvial Gleyic-Colluvic Regosols (YK).

**Additional table 4:** Differential abundance of N cycle gene abundances ratios summarized at the operon level based on linear models and estimate marginal means between soils along the erosion catena.

| **contrast** | **estimate** | **SE** | **df** | **lower.CL** | **upper.CL** | **t.ratio** | **p.value** | **gene** | **int** |
| --- | --- | --- | --- | --- | --- | --- | --- | --- | --- |
| RZ - YK | 1.672 | 0.426 | 23 | 0.441 | 2.903 | 3.920 | 0.002 | aoaaob | pre |
| eLL - LL | 1.243 | 0.461 | 23 | -0.087 | 2.572 | 2.697 | 0.019 | aoaaob | pre |
| eLL - YK | 2.633 | 0.461 | 23 | 1.303 | 3.962 | 5.715 | 0.000 | aoaaob | pre |
| LL - YK | 1.390 | 0.426 | 23 | 0.159 | 2.621 | 3.259 | 0.007 | aoaaob | pre |
| RZ - eLL | 1.096 | 0.372 | 23 | 0.024 | 2.169 | 2.951 | 0.009 | nirknos | pre |
| RZ - LL | 1.688 | 0.344 | 23 | 0.695 | 2.680 | 4.906 | 0.000 | nirknos | pre |
| RZ - YK | 2.810 | 0.344 | 23 | 1.817 | 3.803 | 8.169 | 0.000 | nirknos | pre |
| eLL - YK | 1.714 | 0.372 | 23 | 0.641 | 2.786 | 4.613 | 0.000 | nirknos | pre |
| LL - YK | 1.122 | 0.344 | 23 | 0.130 | 2.115 | 3.263 | 0.005 | nirknos | pre |
| RZ - eLL | 1.329 | 0.192 | 23 | 0.776 | 1.882 | 6.938 | 0.000 | nirsnos | pre |
| RZ - LL | 1.950 | 0.177 | 23 | 1.439 | 2.462 | 10.997 | 0.000 | nirsnos | pre |
| RZ - YK | 2.620 | 0.177 | 23 | 2.108 | 3.132 | 14.773 | 0.000 | nirsnos | pre |
| eLL - LL | 0.621 | 0.192 | 23 | 0.068 | 1.174 | 3.243 | 0.004 | nirsnos | pre |
| eLL - YK | 1.291 | 0.192 | 23 | 0.738 | 1.844 | 6.739 | 0.000 | nirsnos | pre |
| LL - YK | 0.670 | 0.177 | 23 | 0.158 | 1.182 | 3.777 | 0.001 | nirsnos | pre |
| RZ - eLL | -0.766 | 0.181 | 23 | -1.287 | -0.244 | -4.239 | 0.000 | nornos | pre |
| RZ - LL | -1.571 | 0.167 | 23 | -2.054 | -1.089 | -9.395 | 0.000 | nornos | pre |
| RZ - YK | -2.261 | 0.167 | 23 | -2.744 | -1.778 | -13.520 | 0.000 | nornos | pre |
| eLL - LL | -0.805 | 0.181 | 23 | -1.327 | -0.284 | -4.458 | 0.000 | nornos | pre |
| eLL - YK | -1.495 | 0.181 | 23 | -2.017 | -0.974 | -8.278 | 0.000 | nornos | pre |
| LL - YK | -0.690 | 0.167 | 23 | -1.173 | -0.207 | -4.125 | 0.000 | nornos | pre |
| RZ - LL | -1.114 | 0.426 | 23 | -2.345 | 0.117 | -2.613 | 0.031 | aoaaob | post |
| eLL - YK | 1.157 | 0.426 | 23 | -0.074 | 2.388 | 2.714 | 0.031 | aoaaob | post |
| LL - YK | 1.826 | 0.426 | 23 | 0.595 | 3.057 | 4.282 | 0.002 | aoaaob | post |
| RZ - YK | 1.539 | 0.344 | 23 | 0.546 | 2.531 | 4.473 | 0.000 | nirknos | post |
| eLL - YK | 1.541 | 0.344 | 23 | 0.549 | 2.534 | 4.481 | 0.000 | nirknos | post |
| LL - YK | 1.755 | 0.344 | 23 | 0.763 | 2.748 | 5.104 | 0.000 | nirknos | post |
| RZ - eLL | 0.518 | 0.177 | 23 | 0.006 | 1.030 | 2.919 | 0.008 | nirsnos | post |
| RZ - LL | 1.178 | 0.177 | 23 | 0.666 | 1.690 | 6.642 | 0.000 | nirsnos | post |
| RZ - YK | 2.392 | 0.177 | 23 | 1.880 | 2.904 | 13.486 | 0.000 | nirsnos | post |
| eLL - LL | 0.660 | 0.177 | 23 | 0.149 | 1.172 | 3.724 | 0.001 | nirsnos | post |
| eLL - YK | 1.874 | 0.177 | 23 | 1.362 | 2.386 | 10.567 | 0.000 | nirsnos | post |
| LL - YK | 1.214 | 0.177 | 23 | 0.702 | 1.726 | 6.843 | 0.000 | nirsnos | post |
| RZ - LL | -2.374 | 0.529 | 23 | -3.901 | -0.846 | -4.486 | 0.001 | nirsk | post |
| RZ - YK | -2.030 | 0.529 | 23 | -3.557 | -0.503 | -3.837 | 0.003 | nirsk | post |
| eLL - LL | -1.518 | 0.529 | 23 | -3.046 | 0.009 | -2.869 | 0.017 | nirsk | post |
| RZ - eLL | -0.856 | 0.167 | 23 | -1.338 | -0.373 | -5.116 | 0.000 | nornos | post |
| RZ - LL | -1.689 | 0.167 | 23 | -2.172 | -1.206 | -10.098 | 0.000 | nornos | post |
| RZ - YK | -2.802 | 0.167 | 23 | -3.285 | -2.320 | -16.756 | 0.000 | nornos | post |
| eLL - LL | -0.833 | 0.167 | 23 | -1.316 | -0.350 | -4.981 | 0.000 | nornos | post |
| eLL - YK | -1.947 | 0.167 | 23 | -2.429 | -1.464 | -11.639 | 0.000 | nornos | post |
| LL - YK | -1.114 | 0.167 | 23 | -1.596 | -0.631 | -6.658 | 0.000 | nornos | post |
| RZ - YK | 1.192 | 0.372 | 27 | 0.133 | 2.250 | 3.205 | 0.007 | aoaaob | average |
| eLL - YK | 1.784 | 0.385 | 27 | 0.688 | 2.880 | 4.634 | 0.000 | aoaaob | average |
| LL - YK | 1.608 | 0.372 | 27 | 0.549 | 2.667 | 4.324 | 0.001 | aoaaob | average |
| RZ - LL | 0.735 | 0.297 | 27 | -0.110 | 1.580 | 2.477 | 0.030 | nirknos | average |
| RZ - YK | 2.174 | 0.297 | 27 | 1.329 | 3.019 | 7.326 | 0.000 | nirknos | average |
| eLL - YK | 1.616 | 0.307 | 27 | 0.741 | 2.490 | 5.259 | 0.000 | nirknos | average |
| LL - YK | 1.439 | 0.297 | 27 | 0.594 | 2.284 | 4.848 | 0.000 | nirknos | average |
| RZ - eLL | 0.922 | 0.182 | 27 | 0.404 | 1.440 | 5.068 | 0.000 | nirsnos | average |
| RZ - LL | 1.564 | 0.176 | 27 | 1.064 | 2.065 | 8.900 | 0.000 | nirsnos | average |
| RZ - YK | 2.506 | 0.176 | 27 | 2.006 | 3.007 | 14.258 | 0.000 | nirsnos | average |
| eLL - LL | 0.642 | 0.182 | 27 | 0.124 | 1.160 | 3.530 | 0.002 | nirsnos | average |
| eLL - YK | 1.584 | 0.182 | 27 | 1.066 | 2.102 | 8.707 | 0.000 | nirsnos | average |
| LL - YK | 0.942 | 0.176 | 27 | 0.441 | 1.442 | 5.358 | 0.000 | nirsnos | average |
| RZ - LL | -1.652 | 0.407 | 27 | -2.810 | -0.495 | -4.065 | 0.002 | nirsk | average |
| RZ - YK | -1.254 | 0.407 | 27 | -2.411 | -0.097 | -3.085 | 0.014 | nirsk | average |
| RZ - eLL | -0.817 | 0.137 | 27 | -1.206 | -0.427 | -5.968 | 0.000 | nornos | average |
| RZ - LL | -1.630 | 0.132 | 27 | -2.006 | -1.254 | -12.332 | 0.000 | nornos | average |
| RZ - YK | -2.532 | 0.132 | 27 | -2.908 | -2.156 | -19.154 | 0.000 | nornos | average |
| eLL - LL | -0.814 | 0.137 | 27 | -1.203 | -0.424 | -5.946 | 0.000 | nornos | average |
| eLL - YK | -1.715 | 0.137 | 27 | -2.105 | -1.326 | -12.537 | 0.000 | nornos | average |
| LL - YK | -0.902 | 0.132 | 27 | -1.278 | -0.525 | -6.822 | 0.000 | nornos | average |

^1^contrasts: RZ-eLL, RZ-LL, RZ-YK, eLL-LL, eLL-YK, LL-YK

Calcaric Regosol (RZ), strongly eroded Nudiargic Luvisol (eLL), non-eroded Calcic Luvisol (LL) and colluvial Gleyic-Colluvic Regosols (YK).

**Additional table 5:** Significant linear regressions of the response in gene abundances summarized at the operon level with EVI, shoot and soil properties.

| operon | predictor | growth stage | Estimate | Std. Error | p.value | R2 |
| --- | --- | --- | --- | --- | --- | --- |
| *gdh* | elevation | post | 0.941 | 0.09 | 5.70E-08 | 0.88 |
| *hao* | elevation | post | -0.772 | 0.17 | 4.60E-04 | 0.57 |
| *nao* | elevation | post | -0.736 | 0.181 | 1.20E-03 | 0.51 |
| *narAB* | elevation | post | -0.943 | 0.089 | 4.40E-08 | 0.88 |
| *narGHJI* | elevation | post | -0.75 | 0.177 | 8.10E-04 | 0.53 |
| *nasAB* | elevation | post | 0.76 | 0.174 | 6.30E-04 | 0.55 |
| *nifDHKW* | elevation | post | 0.755 | 0.175 | 7.30E-04 | 0.54 |
| *norBC* | elevation | post | -0.902 | 0.115 | 1.80E-06 | 0.8 |
| *nxrAB* | elevation | post | 0.758 | 0.174 | 6.70E-04 | 0.54 |
| *gdh* | EVI | post | -0.935 | 0.094 | 1.00E-07 | 0.87 |
| *gln* | EVI | post | -0.736 | 0.181 | 1.20E-03 | 0.51 |
| *hao* | EVI | post | 0.778 | 0.168 | 3.90E-04 | 0.58 |
| *nao* | EVI | post | 0.76 | 0.174 | 6.40E-04 | 0.55 |
| *narAB* | EVI | post | 0.956 | 0.079 | 7.90E-09 | 0.91 |
| *narGHJI* | EVI | post | 0.779 | 0.168 | 3.80E-04 | 0.58 |
| *nasAB* | EVI | post | -0.782 | 0.167 | 3.50E-04 | 0.58 |
| *nifDHKW* | EVI | post | -0.749 | 0.177 | 8.40E-04 | 0.53 |
| *norBC* | EVI | post | 0.905 | 0.114 | 1.50E-06 | 0.81 |
| *nrfABCD* | EVI | post | -0.752 | 0.176 | 7.70E-04 | 0.54 |
| *nxrAB* | EVI | post | -0.795 | 0.162 | 2.30E-04 | 0.61 |
| *nao* | STC | post | -0.755 | 0.175 | 7.20E-04 | 0.54 |
| *narAB* | STC | post | -0.748 | 0.177 | 8.60E-04 | 0.53 |
| *narGHJI* | STC | post | -0.738 | 0.18 | 1.10E-03 | 0.51 |
| *nasAB* | STC | post | 0.802 | 0.16 | 1.90E-04 | 0.62 |
| *gdh* | SFW | post | -0.751 | 0.177 | 8.00E-04 | 0.53 |
| *nao* | STN | post | 0.799 | 0.161 | 2.00E-04 | 0.61 |
| *narAB* | STN | post | 0.845 | 0.143 | 3.70E-05 | 0.69 |
| *narGHJI* | STN | post | 0.814 | 0.155 | 1.20E-04 | 0.64 |
| *nasAB* | STN | post | -0.775 | 0.169 | 4.20E-04 | 0.57 |
| *nirS* | STN | post | -0.738 | 0.18 | 1.10E-03 | 0.51 |
| *nmo* | STN | post | 0.761 | 0.173 | 6.20E-04 | 0.55 |
| *nrfABCD* | STN | post | -0.827 | 0.15 | 7.70E-05 | 0.66 |
| *nxrAB* | STN | post | -0.802 | 0.16 | 1.90E-04 | 0.62 |
| *amoABC_AOA* | soil.ammonium | post | -0.877 | 0.129 | 8.30E-06 | 0.75 |
| *asn* | soil.ammonium | post | -0.736 | 0.181 | 1.10E-03 | 0.51 |
| *gln* | soil.ammonium | post | -0.857 | 0.138 | 2.30E-05 | 0.71 |
| *gs* | soil.ammonium | post | 0.952 | 0.081 | 1.30E-08 | 0.9 |
| *nao* | soil.ammonium | post | 0.77 | 0.171 | 4.90E-04 | 0.56 |
| *napABC* | soil.ammonium | post | -0.749 | 0.177 | 8.40E-04 | 0.53 |
| *narAB* | soil.ammonium | post | 0.813 | 0.156 | 1.30E-04 | 0.64 |
| *narGHJI* | soil.ammonium | post | 0.808 | 0.157 | 1.50E-04 | 0.63 |
| *nasAB* | soil.ammonium | post | -0.8 | 0.16 | 2.00E-04 | 0.61 |
| *nirK* | soil.ammonium | post | -0.837 | 0.146 | 5.20E-05 | 0.68 |
| *nirS* | soil.ammonium | post | -0.885 | 0.124 | 5.10E-06 | 0.77 |
| *nmo* | soil.ammonium | post | 0.905 | 0.114 | 1.40E-06 | 0.81 |
| *nrfABCD* | soil.ammonium | post | -0.875 | 0.129 | 9.20E-06 | 0.75 |
| *nxrAB* | soil.ammonium | post | -0.934 | 0.095 | 1.20E-07 | 0.86 |
| *gdh* | soil.moisture | post | -0.935 | 0.094 | 1.10E-07 | 0.87 |
| *narAB* | soil.moisture | post | 0.851 | 0.141 | 3.00E-05 | 0.7 |
| *nifDHKW* | soil.moisture | post | -0.875 | 0.129 | 9.10E-06 | 0.75 |
| *norBC* | soil.moisture | post | 0.921 | 0.104 | 4.00E-07 | 0.84 |
| *ans* | elevation | pre | -0.761 | 0.18 | 9.90E-04 | 0.55 |
| *gdh* | elevation | pre | 0.855 | 0.144 | 4.90E-05 | 0.71 |
| *gln* | elevation | pre | 0.796 | 0.168 | 3.90E-04 | 0.61 |
| *narAB* | elevation | pre | -0.913 | 0.113 | 2.10E-06 | 0.82 |
| *narGHJI* | elevation | pre | -0.765 | 0.179 | 9.00E-04 | 0.55 |
| *nasAB* | elevation | pre | 0.802 | 0.166 | 3.20E-04 | 0.62 |
| *nifDHKW* | elevation | pre | 0.799 | 0.167 | 3.60E-04 | 0.61 |
| *norBC* | elevation | pre | -0.737 | 0.188 | 1.70E-03 | 0.51 |
| *nrfABCD* | elevation | pre | 0.735 | 0.188 | 1.80E-03 | 0.51 |
| *gdh* | EVI | pre | -0.847 | 0.147 | 6.70E-05 | 0.7 |
| *gln* | EVI | pre | -0.819 | 0.159 | 1.90E-04 | 0.65 |
| *nao* | EVI | pre | 0.754 | 0.182 | 1.20E-03 | 0.54 |
| *narAB* | EVI | pre | 0.922 | 0.107 | 9.90E-07 | 0.84 |
| *narAB* | EVI | pre | 0.922 | 0.107 | 9.90E-07 | 0.84 |
| *narGHJI* | EVI | pre | 0.793 | 0.169 | 4.20E-04 | 0.6 |
| *nasAB* | EVI | pre | -0.836 | 0.152 | 1.00E-04 | 0.68 |
| *nifDHKW* | EVI | pre | -0.785 | 0.172 | 5.30E-04 | 0.59 |
| *nrfABCD* | EVI | pre | -0.773 | 0.176 | 7.40E-04 | 0.57 |
| *nifDHKW* | SFW | pre | -0.737 | 0.188 | 1.70E-03 | 0.51 |
| *narAB* | STN | pre | 0.738 | 0.187 | 1.70E-03 | 0.51 |
| *amoABC_AOA* | RFW | pre | 0.833 | 0.153 | 1.20E-04 | 0.67 |
| *gln* | V | pre | 0.76 | 0.18 | 1.00E-03 | 0.55 |
| *gs* | RFW | pre | -0.852 | 0.145 | 5.50E-05 | 0.7 |
| *napABC* | RFW | pre | 0.766 | 0.178 | 8.70E-04 | 0.56 |
| *nirK* | RFW | pre | 0.803 | 0.165 | 3.20E-04 | 0.62 |
| *nmo* | RFW | pre | -0.811 | 0.162 | 2.50E-04 | 0.63 |
| ^1^ shoot fresh weight (SFW), root fresh weight (RFW), shoot dry weight (SDW), shoot total carbon (STC), shoot total nitrogen (STN), enhanced vegetation index (EVI). | | | | | |  |

## Supplementary figures


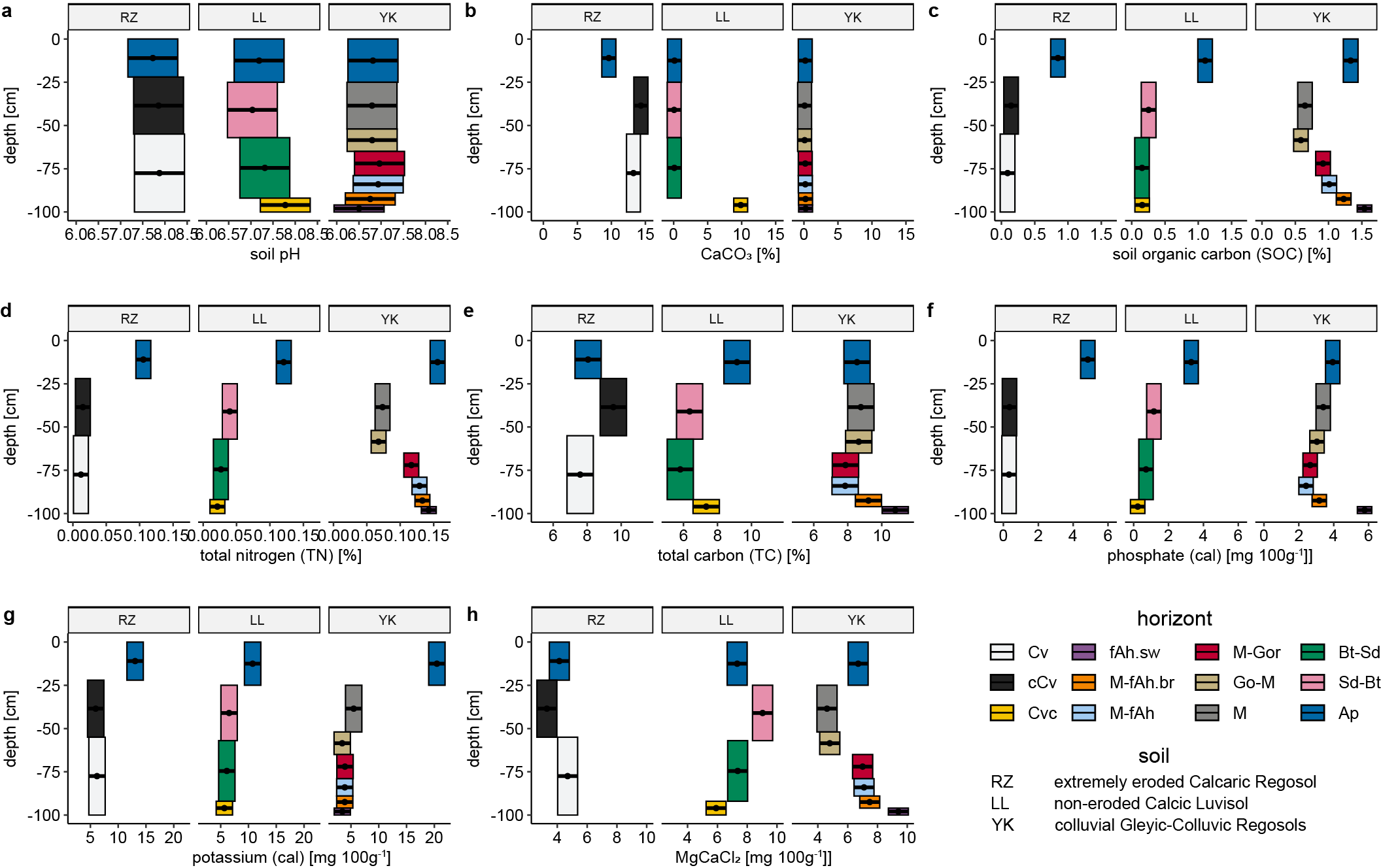


**Additional figure 1.** Parameters of three soils along a tillage erosion catena (1m cores, n=1).


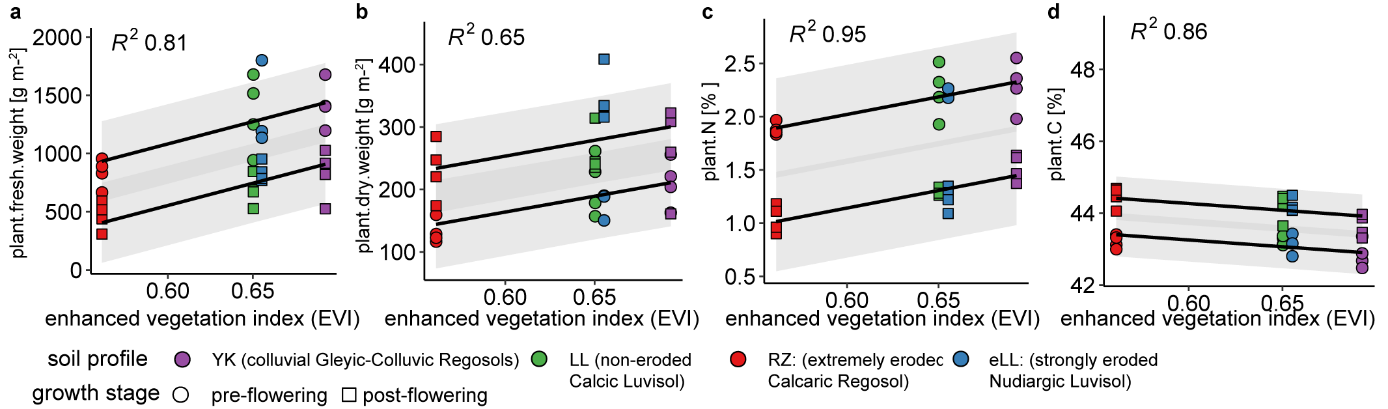


**Additional figure 2.** Linear mixed regression of the response of crop productivity parameters to the EVI as predictor with growth stages as a random factor.


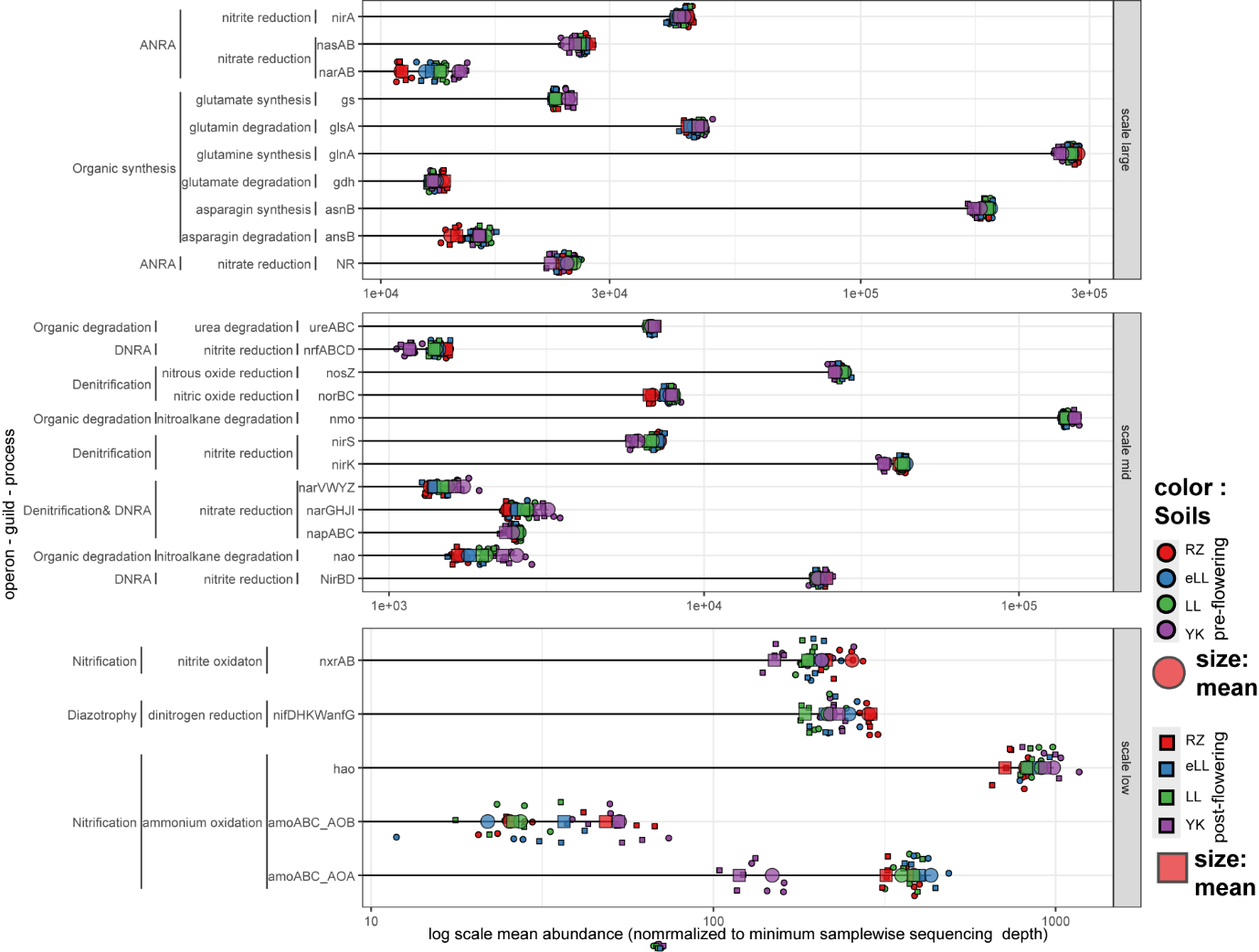


**Additional figure 3.** Relative abundances of N cycling genes at soils^1^ along an erosion catena summarized at the operon level. ^1^extremely eroded Calcaric Regosol (RZ), strongly eroded nudiargic Luvisol (eLL), non-eroded Calcic Luvisol (LL) and colluvial Gleyic-Colluvic Regosols (YK)


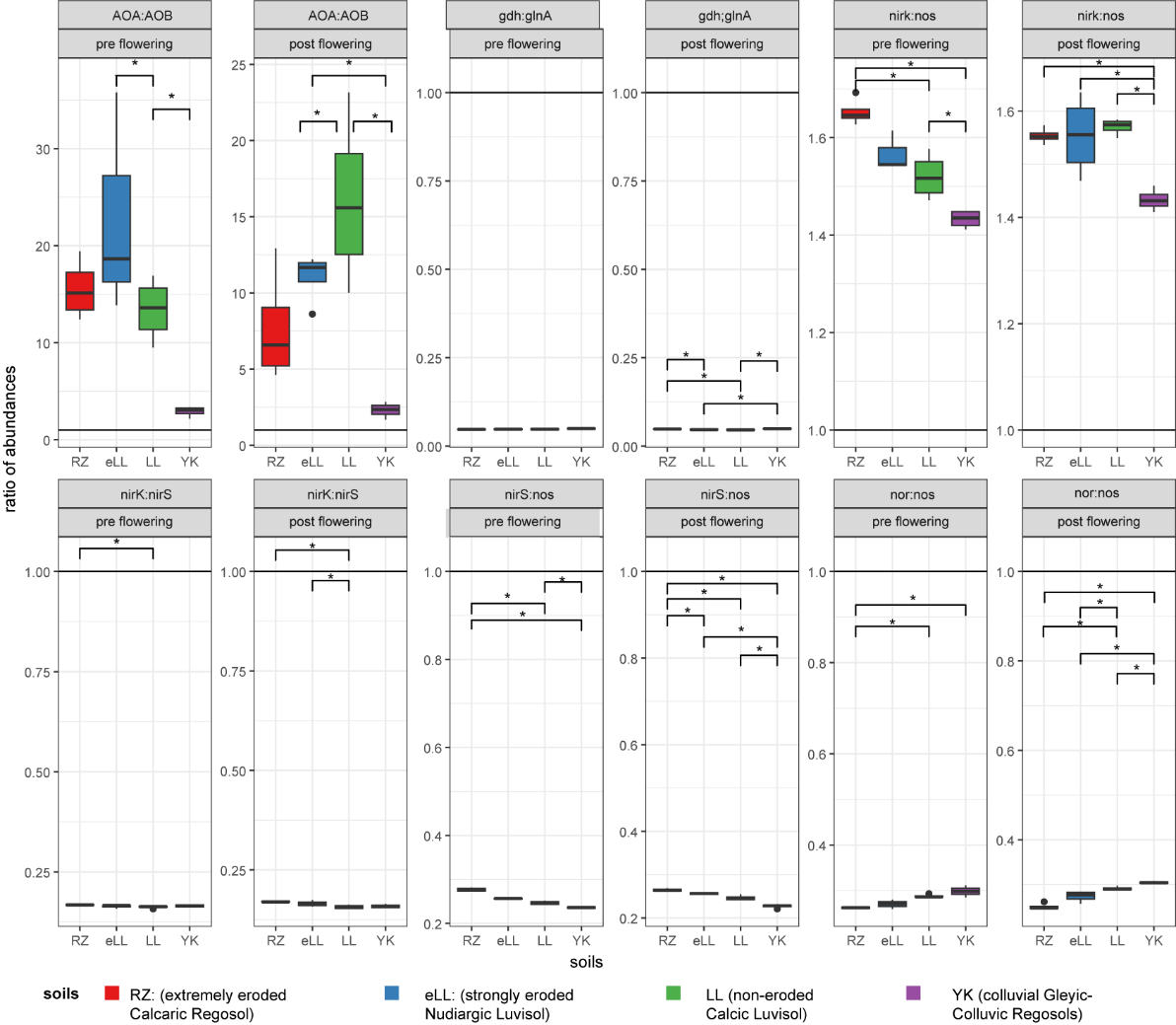


**Additional figure 4.** Relative abundances of ratios between N cycling genes summarized at the operon level. Significance assessment based on linear models and estimate marginal means between soils along the erosion catena.


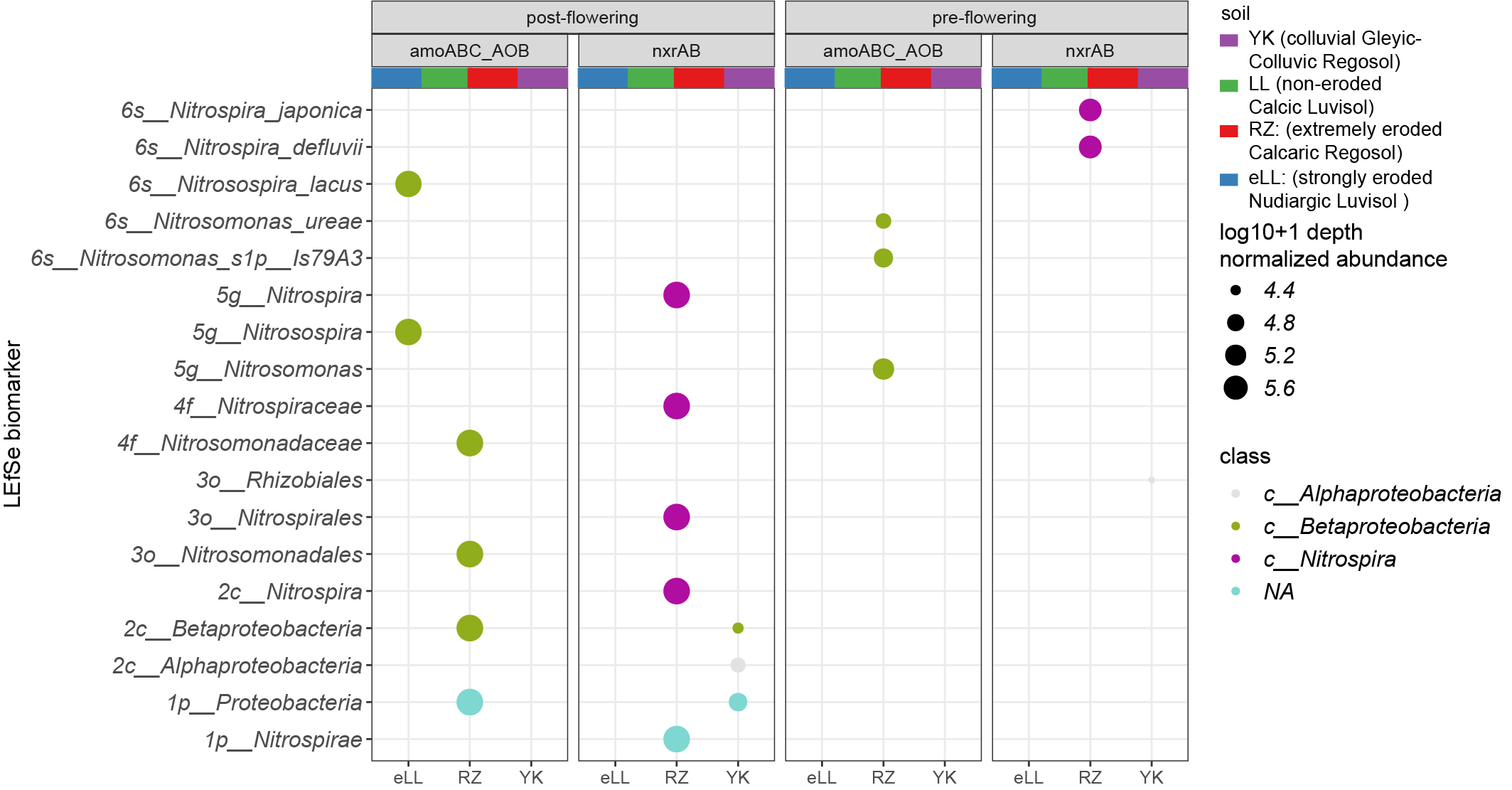


**Additional figure 5:** LEfSe Biomarkers based on kraken2 taxonomic classification of reads annotated to nitrifier guilds in NCycDB.


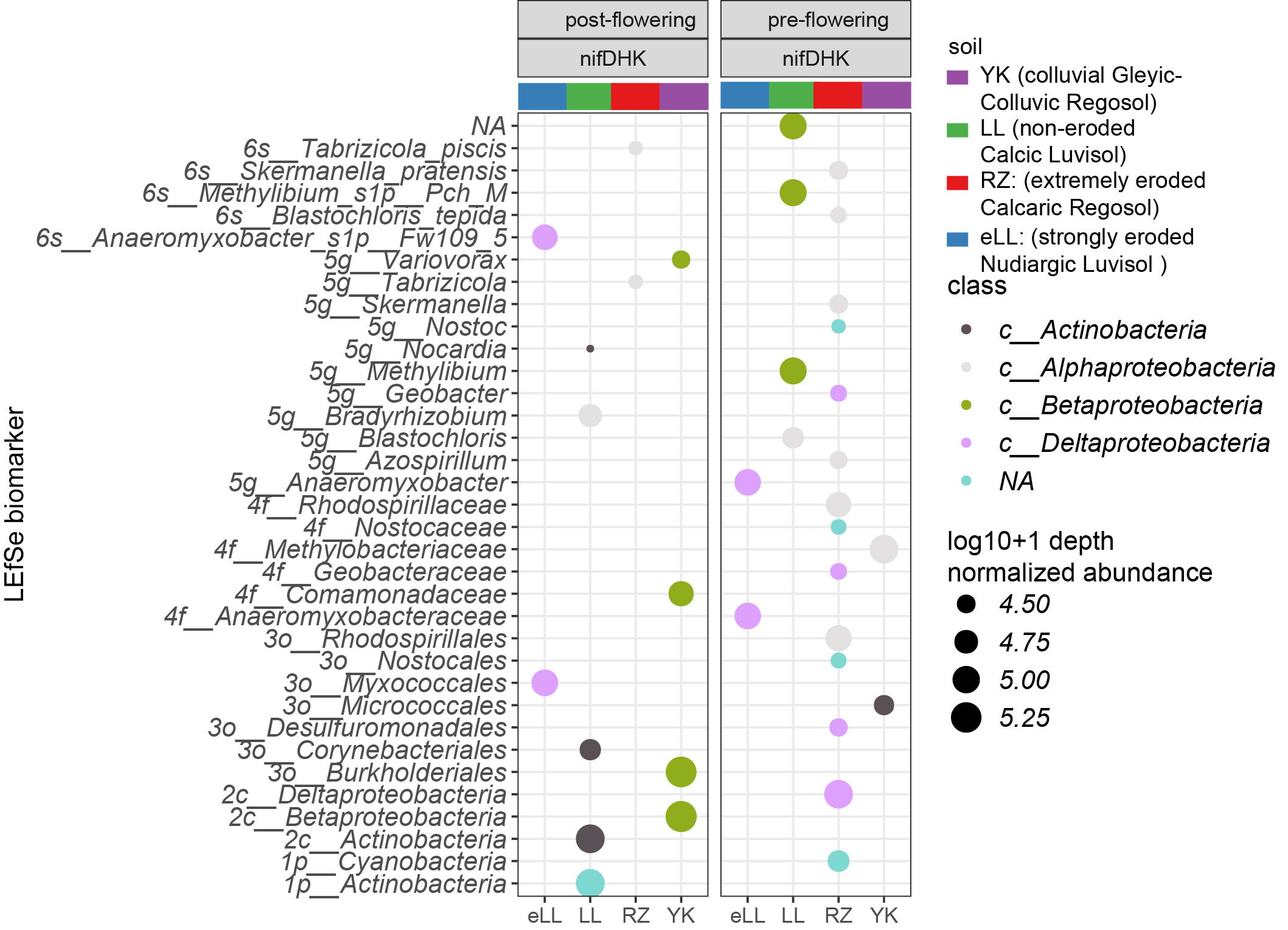


**Additional figure 6:** LEfSe Biomarker based on kraken2 taxonomic classification of reads annotated to diazotoph guilds in NCycDB.


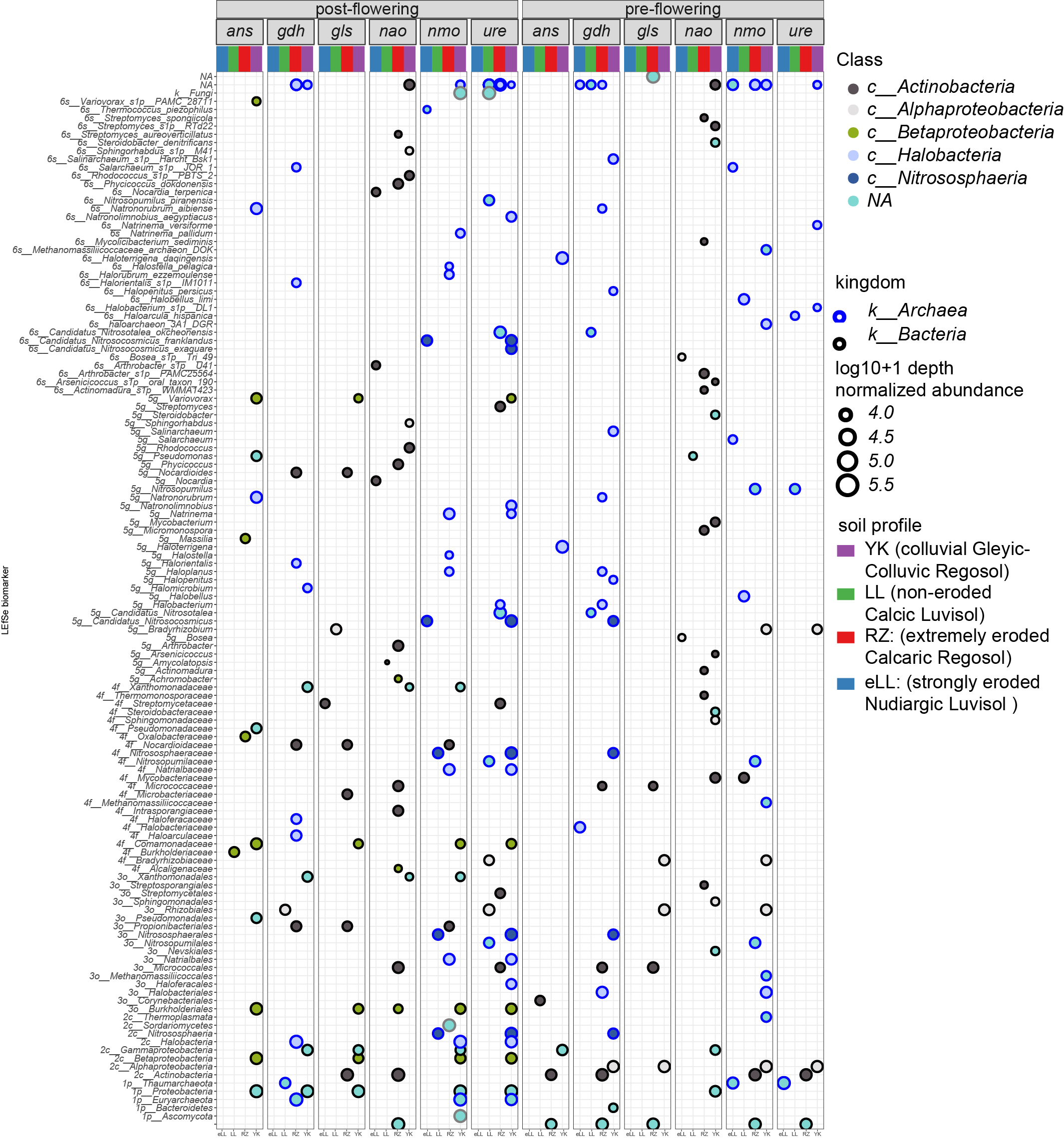


**Additional figure 7:** LEfSe Biomarker based on kraken2 taxonomic classification of reads annotated to genes which encode degradation of organic N compounds based on NCycDB.
